# Determinants for the use and de-implementation of low-value care in health care: a scoping review

**DOI:** 10.1186/s43058-021-00110-3

**Published:** 2021-02-04

**Authors:** Hanna Augustsson, Sara Ingvarsson, Per Nilsen, Ulrica von Thiele Schwarz, Irene Muli, Jessica Dervish, Henna Hasson

**Affiliations:** 1 Procome Research Group, Medical Management Centre, Department of Learning, Informatics, Management and Ethics, Karolinska Institutet, SE 171 77 Stockholm, Sweden; 2 Unit for Implementation and Evaluation, Center for Epidemiology and Community Medicine (CES), Stockholm Region, SE 171 29 Stockholm, Sweden; 3 Department of Health, Medical and Caring Sciences, Division of Society and Health, Linköping University, Linköping, Sweden; 4 School of Health, Care and Social Welfare, Mälardalen University, Box 883, 721 23 Västerås, Sweden

**Keywords:** De-adoption, Disinvestment, De-implementation, Low-value care, Evidence-based

## Abstract

**Background:**

A considerable proportion of interventions provided to patients lack evidence of their effectiveness. This implies that patients may receive ineffective, unnecessary or even harmful care. However, despite some empirical studies in the field, there has been no synthesis of determinants impacting the use of low-value care (LVC) and the process of de-implementing LVC.

**Aim:**

The aim was to identify determinants influencing the use of LVC, as well as determinants for de-implementation of LVC practices in health care.

**Methods:**

A scoping review was performed based on the framework by Arksey and O’Malley. We searched four scientific databases, conducted snowball searches of relevant articles and hand searched the journal *Implementation Science* for peer-reviewed journal articles in English. Articles were included if they were empirical studies reporting on determinants for the use of LVC or de-implementation of LVC. The abstract review and the full-text review were conducted in duplicate and conflicting decisions were discussed until consensus was reached. Data were charted using a piloted data charting form and the determinants were inductively coded and categorised in an iterative process conducted by the project group.

**Results:**

In total, 101 citations were included in the review. Of these, 92 reported on determinants for the use of LVC and nine on determinants for de-implementation. The studies were conducted in a range of health care settings and investigated a variety of LVC practices with LVC medication prescriptions, imaging and screening procedures being the most common. The identified determinants for the use of LVC as well as for de-implementation of LVC practices broadly concerned: patients, professionals, outer context, inner context, process and evidence and LVC practice. The results were discussed in relation to the Consolidated Framework for Implementation Research.

**Conclusion:**

The identified determinants largely overlap with existing implementation frameworks, although patient expectations and professionals’ fear of malpractice appear to be more prominent determinants for the use and de-implementation of LVC. Thus, existing implementation determinant frameworks may require adaptation to be transferable to de-implementation. Strategies to reduce the use of LVC should specifically consider determinants for the use and de-implementation of LVC.

**Registration:**

The review has not been registered.

**Supplementary Information:**

The online version contains supplementary material available at 10.1186/s43058-021-00110-3.

Contributions to the literature

- The study contributes the first synthesis of determinants, for the use of LVC as well as the de-implementation of LVC identified in the peer-reviewed literature.
- The study discusses similarities and differences between determinants for de-implementation and determinants for implementation in relation to the Consolidated Framework of Implementation Research (CFIR) and proposes that implementation determinant frameworks are useful also for de-implementation but may require some adaptations.
- Gaps in the knowledge concerning determinants for the use of LVC and de-implementation of LVC are identified and suggestions for further research are made.

## Background

Use of health care practices (e.g. interventions, programmes and services) with little or no benefit to patients is a widespread problem [1]. Such practices are referred to as low-value care (LVC), which is “care that is unlikely to benefit the patient given the harms, cost, available alternatives, or preferences of the patient” [2]). Estimates show that 12–15% of patients receive at least one LVC practice a year [3] and 72% of US physicians stated that they normally prescribe unnecessary tests or procedures at least once a week [4].

The Choosing Wisely® campaign has produced over 550 recommendations for practices that are considered LVC, including use of antibiotics for upper respiratory infections, imaging for nonspecific low back pain and vitamin D testing [5]. Yet, practices identified as LVC in some clinical circumstances might be of high value in others. Thus, a key challenge is that interventions proven to be effective for specific patient populations are inappropriately applied to patients for whom benefit has never been demonstrated [6].

The use of LVC is costly. For example, the annual cost of LVC for the US Medicare population was estimated to be $8.5 billion, which is almost 3% of total Medicare spending [7]. LVC is a concern both for individual patients and health care systems, and in order to provide evidence-based care to patients, there is a need to de-implement the use of LVC in addition to implementing evidence-based practices. De-implementation involves a structured and planned process using a set of activities to reduce or stop the use of LVC [8]. However, despite dissemination of numerous lists of LVC practices that should be abandoned, e.g. in the Choosing Wisely® campaign [9], the problem with LVC persists. This indicates that lists identifying LVC are not sufficient for these practices to be de-implemented [10].

There is a need to better understand the challenges of de-implementation, including what determinants influence the use of LVC and de-implementation of LVC practices. Determinants of implementation are well known from the current literature [11]. A number of determinants have been described in numerous frameworks, e.g. Consolidated Framework for Implementation Research (CFIR) [12], Promoting Action on Research Implementation in Health Services [13] and Theoretical Domains Framework [14]. These frameworks encompass determinants at multiple levels, from the individual professional, team and department to the organisational and societal levels. However, whether these frameworks are also applicable to categorise determinants of LVC use or de-implementing LVC practices is largely unknown since knowledge is lacking concerning the extent to which these determinants are the same as those pertaining to the implementation of evidence-based interventions [8, 15].

A recent review identified five studies that presented theories, models or frameworks specifically developed for de-implementation of LVC [16]. Four of these [17–20] encompassed determinants that might influence de-implementation of LVC. These determinants had been identified from an existing theory [17], from a literature review on social or behavioural constructs relevant to the de-implementation [20] and from empirical studies [18, 19]. None of these four studies was based on a literature review of determinants for de-implementation; thus, determinants for de-implementation of LVC has not been synthesised. Categorisations of determinants for the use of LVC and de-implementation of LVC could benefit the development of frameworks for de-implementation as well as adaptations of existing implementation frameworks to ascertain their applicability for de-implementation of LVC. Such a synthesis could also be important for exploring different strategy options to address the determinants to achieve de-implementation of LVC. Therefore, the aim of this review was to (1) identify and categorise determinants influencing the use of LVC and (2) identify and categorise determinants for de-implementation of LVC practices in health care. The identified determinants are discussed in relation to CFIR.

## Methods

### Design

We performed a scoping review methodology because it lends itself to address broader topics where studies using different designs and methods are synthesised [21]. The review process was based on the framework by Arksey and O’Malley [21], which outlines five stages: (1) identifying the research question, (2) identifying relevant studies, (3) study selection, (4) charting the data and (5) collating, summarising and reporting the results. The review is reported according to the PRISMA-ScR Checklist [22] (Additional file 1.).

### Protocol and registration

The methodology has been described in a study protocol outlining the research project [23].

### Eligibility criteria

We included empirical studies written in English and published in a peer-reviewed journal. Using the PCC mnemonic (Population, Concept, Context), recommended for scoping reviews [24], we specified that the content of the citations should describe determinants for the use of LVC practices and/or the de-implementation of LVC practices (concept) within health care (context). We did not specify a population since we were interested in determinants for the use and de-implementation of LVC in health care in general. All types of study designs were included, with the implication that studies reporting on supposed associations based on qualitative and descriptive data as well as associations based on quantitative data and statistical tests were included. To be included, a study needed to refer to a formal recommendation or a guideline stating that the practice was not recommended for a certain population/setting, i.e. of low value, to avoid judgements by the authors. We excluded studies that investigated determinants for potential overuse of a practice without identifying that the use was in fact inappropriate. All eligibility criteria are reported in Table 1.

**Table 1:** Eligibility criteria for inclusion

1. English language	
2. Published between January 2013 and June 2018	
3. Published in a peer-reviewed journal	
4. Empirical study	
5. **P**opulation: not specified	
6. **C**oncept: Determinants for the *use of LVC*, Determinants for *de-implementation of LVC* (NB. For studies about determinants for the *use of* LVC the study needed to refer to a recommendation [e.g. choosing wisely] or a guideline [e.g. clinical guidelines] stating that the practice is not recommended)	
7. **C**ontext: Health care setting (including primary care, hospital care, community care and mental health)	

### Information sources

Searches were done in four electronic databases from different disciplines considered to be relevant to the topic of LVC and de-implementation, such as biomedicine, health services research and nursing (MEDLINE, Embase, CINAHL and Web of Science). We also conducted hand searches of *Implementation Science* and of reference lists from relevant articles identified in the abstract screening.

### Search

Potential keywords were identified through discussions among the authors, discussions with the Swedish Agency for Health Technology Assessment and Assessment of Social Services, from a review outlining terminology used for de-implementation [25] and through inspection of key papers focusing on de-implementation. The keywords were integrated into a search strategy in collaboration with librarians at the Karolinska Institutet library. The search terms were tested and refined three times to ensure that they captured 18 identified example papers and were discriminant enough to avoid yielding an overwhelming amount of citations. The librarians searched the databases from January 1, 2013, to June 4, 2018. This time limit was chosen because database searches showed an increasing number of papers from 2013 and onwards and due to a need to limit the number of studies since a vast amount of irrelevant studies was captured. The search strategy used in Web of Science is detailed in Table 2. Search strategies for all databases are included as an attachment (Additional file 2).

**Table 2:** Search strategy used in Web of Science

Field labels	
• TS/Topic = title, abstract, author keywords and Keywords Plus	
• NEAR/x = within x words, regardless of order	
• * = truncation of word for alternate endings	
#1 TOPIC: (((abandon* OR contradict* OR deadopt* OR ”de-adopt*” OR disadopt* OR ”dis-adopt*” OR decommission* OR ”de-commission*” OR deimplement* OR ”de-implement*” OR delist* OR ”de-list*” OR disinvest* OR ”dis-invest” OR deprescript* OR deprescrib* OR divest* OR inapprop* OR ineffective* OR ”low-value” OR obsole* OR outmoded OR overuse OR reallocate* OR reassess* OR ”re-assess*” OR refute* OR refuting OR ”re-invest*” OR ”medical revers*” OR supersed* OR unlearn*) NEAR/3 (care OR clinic* OR device* OR drug OR drugs OR evidence* OR health OR healthcare OR medical OR medication* OR prescrib* OR procedur* OR technolog* OR therap* OR treat*)))	
#2 (((chang* or discontinu* or ”dis-continu*” or decreas* or declin* or drop or reduc* or withdraw*) NEAR/1 ("use" or practice) NEAR/3 (care or clinic* or device* or drug or drugs or evidence* or health or healthcare or medical or medication* or prescrib* or procedur* or technolog* or therap* or treat*)))	
#3 TS=("choosing wisely" or "priority setting") AND TS=(care or clinic* or device* or drug or drugs or evidence* or health or healthcare or medical or medication* or prescrib* or procedur* or technolog* or therap* or treat*) #4 #3 OR #2 OR #1	
#5 ((abandon* OR contradict* OR deadopt* OR ”de-adopt*” OR disadopt* OR ”dis-adopt*”	
OR decommission* OR ”de-commission*” OR deimplement* OR ”de-implement*” OR delist* OR ”de-list*” OR disinvest* OR ”dis-invest” OR discontinu* OR ”dis-continu*” OR deprescipt* OR deprescrib* OR divest* OR inapprop* OR ineffective* OR ”low-value” OR obsole* OR outmoded OR overuse OR reallocate* OR reassess* OR ”re-assess*” OR refute* OR refuting OR ”re-invest*” OR ”medical revers*” OR supersed* OR unlearn* OR withdraw*) NEAR/3 (factor* OR barrier* OR engag* OR ”evidence-based” OR facilitat* OR determinant* OR predict* OR model* OR framework* OR intervent* OR policy OR policies OR ”practice pattern*” OR program* OR strateg* OR tool*))	
#6 #5 AND #4	
Refined by: LANGUAGES: ( ENGLISH )	

### Selection of sources of evidence

All identified citations were imported into Rayyan, a web-based and mobile app that organises and facilitates the initial screening of titles and abstracts as well as the collaboration between reviewers [26]. The eligibility criteria were tested in multiple steps to ensure consensus among the reviewers. As a first step, four reviewers (HA, SI, IM, JD) tested the criteria on a sample of 40 abstracts. Inconsistencies were discussed and clarifications of the criteria were made. As a second step, reviewers piloted an additional 40 abstracts, which resulted in minor refinements of the criteria. Thereafter, five reviewers (HA, SI, IM, JD, PN) applied the eligibility criteria to all citations. All abstracts were assessed independently by two reviewers. When all abstracts had been screened, the reviewers discussed the conflicting decisions. In cases where disagreement or uncertainty existed, the whole reviewer group discussed until a consensus was reached.

Thereafter, the five reviewers assessed the full texts of the included citations for final inclusion. This was done in pairs so that all articles were assessed independently by two reviewers. Conflicting decisions were discussed between the two reviewers, and in case of uncertainty, the article was discussed among the full reviewer group.

### Data charting

A data charting form was developed. Five of the authors (HA, SI, IM, JD, PN) piloted the form by independently extracting data from three studies. The data were compared and inconsistencies were discussed, resulting in some changes of the form. A second test was made, in which the authors independently extracted data from two additional studies, which resulted in minor refinements of the form. The five authors then independently extracted data from the rest of the studies and continuously held meetings about any difficulties that emerged. Difficulties that were unable to be resolved were discussed by all authors. One of the authors (HA) read the extracted data from all 101 included studies to look for potential inconsistencies in what data had been extracted. This resulted in some minor additions of information for a few of the included studies.

### Data items

Data charted related to general information about the citations (e.g. author) as well as to data related to the study aim (e.g. determinants for de-implementation) (Table 3). The first authors’ affiliations were used as a proxy for country study origin when information about origin was missing.

**Table 3:** Items for data charting

Overview of items for data charting	
a) Title	
b) Journal	
c) Authors	
d) Year published	
e) Country of study origin	
f) Stated aim	
g) Type of healthcare setting	
h) Qualitative, quantitative, mixed methods	
i) Study design	
j) Method to assess the determinants (e.g. interviews, record review, survey)	
k) Study participants (type and number)	
l) Type of LVC practice	
m) Guideline or recommendation	
n) De-implementation determinants	
o) Use of LVC determinants	

### Synthesis of results

An overview of the extent, nature and distribution of the included studies was reported in tables and text. Content analysis was conducted to inductively code and categorise the extracted information about the determinants of LVC and de-implementation of LVC. Inductive analyses is preferred when the knowledge base is limited [27]. Thus, the reason for applying an inductive approach instead of a deductive coding based on an existing implementation framework was that the knowledge about determinants for use of LVC and de-implementation of LVC is scarce and the extent to which determinants overlap with implementation determinants is unknown [8, 28]. Using an inductive approach assured that determinants were identified, coded and categorised without the preconception that they were the same as for implementation. The coding entailed reading the extracted data and providing a code for the meaning of the determinants. For example, “cardiologists ordered inappropriate transthoracic echocardiography more frequently than other specialties” was assigned the code *professional specialty*. The coding of each determinant was conducted by at least two authors. Five of the authors (HA, SI, PN, UvTS, HH) then categorised the codes by scrutinising each individual code and sorting codes related to the same type of determinant into subcategories. For instance, the code *professional specialty*, defined as the health care professionals’ medical specialisation, was sorted into the subcategory professional characteristics which address age, gender, years of experience in the profession, professional specialisation, medical school affiliation, lack of experience and personality. Thereafter, the subcategories were clustered into broader categories, e.g. the subcategory professional characteristics was sorted into a professional determinant category. All identified categories, subcategories and individual codes are outlined in Additional file 3.

Frequencies were used to summarise the number of studies reporting on specific determinants within the subcategories and categories. Information about the direction of the influence of the determinants, as reported in the included studies (both qualitative and quantitative studies), were indicated by referring to whether the determinant contributed to LVC use or was associated with lower use of LVC. The direction of the influence of determinants for de-implementation was indicated by specifying whether the determinant acted as a facilitator or barrier to de-implementation of LVC. The strengths of associations were not taken into account or reported since the scoping review encompassed studies with designs that do not allow for statistical tests of the associations, including qualitative studies. The synthesis and reporting of results were divided into two parts: (1) determinants for *the use of LVC* practices and (2) determinants for *de-implementation of LVC*, i.e. a structured and planned process using a set of activities to reduce or stop the use of LVC.

## Results

### Study selection

**Fig. 1:**
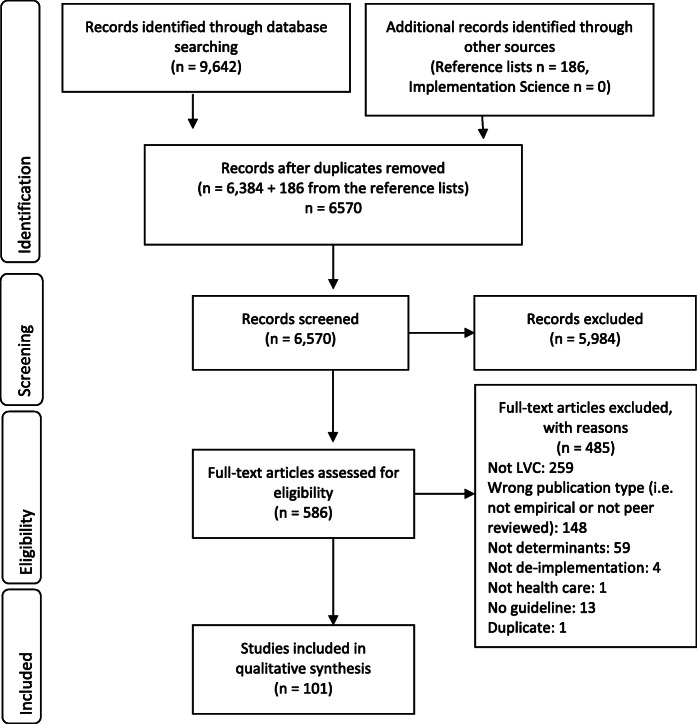
PRISMA flow diagram

The database search yielded 9542 citations (Fig. 1). An additional 186 citations were identified through searches in reference lists. After removing duplicates, 6570 citations remained for abstract screening. Of these, 586 citations were included for assessment in the full-text review. In total, 101 citations were included in the final review.

### Study characteristics

Of the 101 studies, 92 focused on determinants for the *use of LVC* and nine studies on determinants for *de-implementation of LVC*. These two types of studies are described separately below.

#### Characteristics of studies on determinants for the use of LVC

The majority of studies were conducted in the USA (*n* = 54) and the most common settings were hospital and primary care (Table 4). Most studies had a quantitative design and collected data through record reviews and surveys. Data were collected from professionals in 28 studies, patients in 51 studies and from professionals as well as patients in 13 studies.

**Table 4:** Study characteristics for the studies investigating determinants for the use of LVC

Study aspect	Number of studies (total ***n*** = 92)	% of total number of studies
**Healthcare setting**		
Hospital	34	36.9
Primary care	24	26.1
Multiple settings	17	18.5
Aged care	7	7.6
Non-specific setting	8	8.7
Other^a^	2	2.2
**Method**		
Quantitative	78	84.8
Qualitative	11	12
Mixed methods	3	3.2
**Study design**		
Retrospective review	40	43.5
Cross-sectional	32	34.8
Cohort	4	4.3
Experimental	2	2.2
Prospective observations	3	3.2
Other^c^	11	12
**Data collection method**^**b**^		
Record review	50	54.3
Survey	30	32.6
Interviews	11	12
Focus groups interviews	3	3.3
Administrative data	2	2.2
**Participants in the studies**		
Healthcare professionals	28	30.5
Patients	51	55.4
Healthcare professionals and patients	13	14.1

^a^Dentistry, Informal healthcare sector

^b^Total percentage exceeds 100% as some studies used more than one data collection method.

^c^Qualitative interview studies

The included studies investigated a range of LVC practices (Table 5). The majority of studies (*n* = 73) focused on one LVC practice. In total, 165 types of LVC practices were investigated.

**Table 5:** Type of LVC practices investigated in the studies examining determinants for the use of LVC

Type of LVC practice	Number of practices (total ***n*** = 165)^a^	% of total number of practices
Medication prescriptions	69	41.8
Imaging	41	24.9
Screening	20	12.1
Cardiovascular testing and imaging	14	8.5
Diagnostic tests	11	6.7
Procedure (non-surgical and surgical)	3	1.8
Radiation therapy	2	1.2
Other^b^	5	3.0

^a^The number of studies exceeds the total number of studies included in the review and the percentage exceeds 100% because some studies investigated several LVC practices

^b^E.g. feeding tubes in dementia patients, non-indicated foley catheter use

The practices were sorted into eight categories. The most common LVC was *medication prescriptions* (*n* = 69), encompassing non-indicated prescription of antibiotics, potentially inappropriate medications and antipsychotics. A total of 41 practices concerned *imaging* (e.g. imaging for back pain) and 20 practices were related to *screening*, including vitamin D deficiency screening for low-risk patients and ECG screening in general medical examinations. Fourteen practices concerned *cardiovascular testing and imaging* and varying *diagnostic tests* (e.g. thyroid function test in asymptomatic patients and unnecessary pre-operative tests) were the focus of 11 studies. The remaining practices concerned *non-surgical and surgical procedures* (*n* = 3) and *radiation therapy* (*n* = 2). Five studies could not be categorised in any of these categories and were labelled *other*.

#### Characteristics of studies on determinants for de-implementation of LVC

Of the nine studies investigating determinants for de-implementation of LVC, five were conducted in the USA (Table 6). Most studies were conducted in primary care and hospital settings. The majority of studies (*n* = 5) had a quantitative design and collected data from professionals (*n* = 7).

**Table 6:** Study characteristics for studies investigating determinants for de-implementation of LVC

Study aspect	Number of studies (total ***n*** = 9)	% of total number of studies
**Healthcare setting**		
Hospital	3	33.3
Primary care	3	33.3
Policy	2	22.2
Anticoagulation clinic	1	11.1
**Method**		
Quantitative	5	55.6
Mixed method	3	33.3
Qualitative	1	11.1
**Study design**		
Cross-sectional	6	66.7
Experimental	1	11.1
Longitudinal	1	11.1
Three-round Delphi	1	11.1
**Data collection method**^**a**^		
Survey	7	77.8
Interviews	3	33.3
Record review	1	11.1
Delphi	1	11.1
**Participants in the studies**		
Healthcare professional	5	55.6
Healthcare professionals and other stakeholders	2	22.2
Patients	2	22.2

^a^Total percentage exceeds 100 %, as some studies used more than one data collection method

Most de-implementation studies focused on single LVC practices (*n* = 4) (Table 7). Two of the studies concerned multiple LVC practices and two investigated LVC practices in general. A total of 21 LVC practices were identified and the most studied type of LVC practice was medication prescriptions (*n* = 7), which included practices such as non-indicated prescription of antibiotics.

**Table 7:** Type of LVC practices investigated in the studies examining determinants for de-implementation of LVC

Type of LVC practice	Number of practices (total ***n*** = 21)	% of total number of practices
Medication prescriptions	7	33.3
Screening	5	23.8
Imaging	4	19
Tests	3	14.3
Procedures	2	9.5

### Determinants for the use of LVC

The identified determinants were related to patients, professionals, outer context, inner context, process and evidence and LVC practice. These categories include a number of subcategories (Table 8). A table with all identified determinants in each study is provided in Additional file 4.

**Table 8:** Identified determinants for the use of LVC and de-implementation of LVC

Studies on determinants for LVC use total ***n*** = 92				Studies on determinants for de-implementation total ***n*** = 9		
Determinants	Total no of studies including the determinant	No (%) of studies linking the determinant to LVC use	No (%) of studies linking the determinant to low LVC use	Total no of studies including the determinant	No (%) of studies with the determinant as a facilitator of de-implementation	No (%) of studies with the determinant as a barrier to de-implementation
**Patient determinants**						
Patient characteristics	39	37 (94.9)	10 (25.6)	-		
Patient health condition	19	17 (89.5)	2 (10.5)	-		
Patient expectations	16	16 (100)		5		5 (100)
Patient knowledge	-			2	1 (50.0)	1 (50.0)
Expectations from relatives	2	2 (100)		-		
**Professional determinants**						
Professional characteristics	28	24 (85.7)	7 (25.0)	-		
Professional knowledge of LVC	9	6 (66.7)	3 (33.4)	1		1 (100)
Professionals’ expectations, attitudes and behaviours	18	17 (94.4)	2 (11.1)	5		5 (100)
Professionals' memory	-			1		1 (100)
**Outer context determinants**						
Location	14	13 (92.9)	5 (35.7)	-		
Economy	15	13 (86.7)	4 (26.7)	1		1 (100)
Outer context characteristics	7	4 (71.4)	3 (42.9)	-		
Patient volume	4	4 (100)		-		
Policy and political support	3	1 (33.4)	2 (66.7)	3	2 (66.7)	1 (33.4)
Marketing	3	3 (100)		-		
Time	4	4 (100)		-		
Pressure from suppliers	-			1	1 (100)	
**Inner context determinants**						
Setting characteristics	19	15 (78.9)	6 (31.6)	1	1 (100)	
Work/care process	20	18 (90.0)	4 (20.0)	3		3 (100)
Staff composition	3	3 (100)		-		
Organisational structures related to the LVC	5	5 (100)		-		
Interaction between professionals	4	4 (100)		1		1 (100)
Culture	3	3 (100)		1	1 (100)	
Patient-provider communication/interaction	2	2 (100)		-		
**Process determinants**						
Strategies	4		4 (100)	-		
De-implementation process	-			2	1 (50.0)	1 (50.0)
**Evidence and LVC practice determinants**						
Evidence	7	7 (100)		1		1 (100)
Characteristics of the LVC	2	2 (100)		-		
Negative consequences of reducing LVC for the professional	-			1		1 (100)
Characteristics of alternative practice	2	2 (100)		1		1 (100)
Other^a^	3	3 (100)		-		

^a^Patient already using the LVC, day of the week (Wednesday), disease label

#### Patient determinants

Patient-related determinants were the most commonly identified type of determinant. The studies reported a total of 72 determinants that contributed to the use of LVC and 12 determinants that contributed to lower use of LVC. The category consists of four subcategories.

*Patient characteristics* were identified as a determinant contributing to the use of LVC in 37 studies and to lower use in 10 studies and included, e.g. age, gender, ethnicity and socio-economic factors. There was no consistent pattern as to the positive or negative influence on LVC. For instance, older age was most often associated with use of LVC (e.g. [29–31]), but there were also studies where younger age was associated with LVC (e.g. [32, 33]). *Patient health condition* was found to contribute to use of LVC in 17 studies and to lower use in two studies. Patient health condition included determinants such as severity of illness (e.g. [34]) and characteristics of the disease (e.g. [35]). *Patient expectations*, e.g. patients who requested non-indicated prescriptions, increased the occurrence of LVC in 16 studies (e.g. [36, 37]). Also *expectations from relatives* (*n* = 2) contributed to the use of LVC [38, 39].

#### Professional determinants

In total, 47 professional determinants contributed to use of LVC and 12 determinants to lower use of LVC. The category included three subcategories.

*Professional characteristics* encompassed determinants such as age, gender, medical speciality, professional training and personality, which were identified in 24 studies to contribute to the use of LVC and found to be related to lower use of LVC in seven studies. As with patient characteristics, the results were inconsistent regarding age, gender and length of experience of the professionals. Lack of or inadequate training was consistently linked to use of LVC (e.g. [40, 41]). *Professionals’ knowledge of LVC* both contributed to the use of LVC (*n* = 6) and protected against LVC use (*n* = 3). Being aware of a guideline advising against the use of the practice was related to less LVC (e.g. [42]), whereas not having access to guidelines (e.g. [43]) or misinterpreting guidelines [44] increased LVC. A lack of knowledge about cost-effectiveness [45] and lack of cost-awareness [46] were linked to the use of LVC.

The majority of determinants in *professionals’ expectations, attitudes and behaviours* were related to higher use of LVC (*n* = 17). Determinants included professionals’ fear of malpractice, which was a commonly identified reason for providing LVC. For instance, the risk of missing a diagnosis was perceived to be more important than the risk of exposing patients to unnecessary radiation in relation to LVC imaging [47]. Another related determinant was fear of liability or litigation as a reason for using LVC, e.g. unnecessary diagnostic tests [48]. Professionals’ expectations, attitudes and behaviours also included professionals’ desire to meet patient requests (e.g. [45, 49]), attitudes toward or beliefs about existing evidence and guidelines (e.g. [50]) and the habit of using a practice (e.g. [46, 51]). Two studies identified determinants that were linked to lower use of LVC: professionals’ cost-consciousness [42] and perceived usefulness of guidelines as well as self-efficacy with regard to discussing LVC and costs [52].

#### Outer context determinants

The outer context included determinants related to the social, political and geographical context of health care, comprising six sub-categories. In total, 42 determinants related to a higher use of LVC and 15 determinants related to lower use were reported.

*Location* referred to the type of geographical area, e.g. metropolitan, urban, suburban and rural as well as different identified regions. There was no clear pattern concerning what type of geographical area was linked to higher or lower use of LVC, i.e. this differed in the included studies (e.g. [33, 50, 53, 54]). *Economy* included determinants contributing to LVC use (*n* = 14) as well as determinants associated with lower use of LVC (*n* = 4) and was related to the financing of health care and financial incentives. In the majority of the studies, having private health insurance was linked to receiving LVC, when compared to other insurance types [53–56]. However, having Medicaid or being uninsured was also associated with receiving LVC [57]. Although inappropriate prescribing measures were more common in uninsured patients and patients with Medicaid, compared to privately insured patients, computed tomography/magnetic resonance imaging for back/neck pain was used to a higher extent for patients with private insurance [58]. The category also included studies on reimbursement models and financial incentives. Incentives for using LVC [48], fee-for-service funding [59] and patients not sufficiently sharing in the cost of their health care [45] were related to a higher use of LVC, and a decreased reimbursement for a LVC practice was associated with lower use of the practice [60].

*Outer context characteristics* included determinants related to the patient or professional population in the specific area for the study. Such characteristics were related to higher as well as lower use of LVC practices in four studies, respectively. For instance, municipalities with lower socioeconomic status [61] and regions with high health care consumption [62] were found to have a higher extent of LVC. A higher specialist to primary care ratio [62] and specialist density [63] were associated with higher use of LVC services, and higher physician group concentration (a measure of market competition) was linked to lower use of LVC practices [62]. *Patient volume* influenced LVC use in four studies. For example, lower patient volume at practice level was related to inappropriate prostate cancer imaging [64] and inappropriate breast cancer imaging as assessed by clinical vignettes [50]. However, when it came to individual physicians’ patient volume, a higher volume of patients per day was related to a higher use of LVC [65].

*Policy and political support* were found to influence the use of LVC in three studies. Having a policy concerning restricted LVC use was linked to lower use [59, 66], and a lack of political support, on the other hand, was perceived to be a barrier to reduce overuse [67]. *Marketing* in the form of direct-to-consumer advertising about drugs and treatments [45], promotion of screening directed to the population [59] and interests of health care companies or the pharmaceutical industry [48] were identified as influencing the use of LVC in three studies. *Time* was addressed in four studies that investigated the fluctuation of LVC use over time, i.e. from 1 year to another. These studies showed that the use of specific LVC practices was inconsistent and increased or decreased from one year to another without any clear trend over time [33, 64, 68, 69].

#### Inner context determinants

The inner context comprised determinants related to the structural and social environments of the health care settings, e.g. hospitals, clinics and care centres, and entailed seven subcategories. Fifty determinants were linked to a higher use of LVC and 10 related to lower use.

*Setting characteristics* included studies that compared the extent of LVC between different types of settings, e. g. hospital-based practices compared with community-based practices (e.g. [70–72]), usually without providing information about potential reasons for the varying use. *Work/care process* included lack of care continuity (e.g*.* number of handoffs and having several care providers) which was linked to a higher use of LVC practices (e.g. [73–76]). Perceived lack of time and time pressure when performing work tasks were associated with higher use of LVC (e.g. [36, 77]). Other identified determinants included perceived inaccessibility of decision support [44], individual rather than clinic-based decision-making [59] and lack of feedback/accountability [49], which all contributed to more LVC. Multidisciplinary rounds [75] and teamwork [40] were found to be related to lower use of LVC. The subcategory *Staff composition* included inadequate staffing levels [40] and solo practice [60, 78] which were linked to higher use of LVC.

*Organisational structures related to the LVC* referred to structures in the organisation that provided incentives for or facilitated using LVC, such as the ownership of equipment that were used for providing the LVC practice [51, 79] and structures that made it easy to order repeating/standing lab tests [46]. *Interaction between professionals* mainly included the recommendation, expectation or request by another professional or specialist to provide the LVC practice [37, 59, 80]. Also, a lack of communication between professionals was identified as a determinant for the use of LVC in one study [44]. Different types of *culture* were identified to contribute to the use of LVC in three studies. A screening culture was considered to influence prostate-specific antigen (PSA) testing in Australia [59], a hierarchical culture with a lack of debate was perceived to contribute to LVC in primary care [48] and a culture of poor cost consciousness was perceived to contribute to unnecessary inpatient laboratory tests [46]. *Patient-provider communication/interaction* was identified as influencing the use of LVC in two studies, describing challenges that care providers faced when communicating with patients about unnecessary antibiotics [43] and a paternalistic doctor-patient relationship that contributed to an environment in which potentially inappropriate prescribing can occur [81].

#### Process determinants

The process involved determinants related to processes for managing LVC and was the least common category reported (*n* = 4). This category included the subcategory *strategies*, which referred to strategies used to limit the use of LVC. The identified strategies were linked to lower use of LVC and included having processes and routines for managing LVC issues (meetings, review and communication with care recipients and relatives) [82]; managerial priorities for non-pharmacological management [40] patient education, provider education and decision support [43]; and medication reviews [41].

#### Evidence and LVC practice determinants

Determinants related to the evidence and the LVC practice were linked to LVC use in 11 studies and included three sub-categories.

*Evidence* included conflicting guidelines [50, 59] and inappropriate or inapplicable guidelines for the patient group [52, 77], which led to more LVC. Furthermore, professionals’ belief that the LVC was, in fact, effective, despite guidelines recommending against its use was reported as a reason for using LVC (e.g. [41]). *Characteristics of the LVC* was a determinant when the LVC practice had advantages, such as being easy to distribute [41] or lacking perceived negative consequences [41, 47]. *Characteristics of alternative practice* (i.e. an alternative to the LVC practice), such as when the alternative lacked guidelines [41] or access [83], was also a determinant for LVC.

### Determinants for de-implementation of LVC

The nine studies concerning determinants for de-implementation of LVC were related to the same broad categories as the determinants for using LVC. Each category consisted of a number of subcategories (Table 8). A table with all identified determinants in each study is provided in Additional file 4.

#### Patient determinants

Patient determinants typically acted as barriers to de-implementation. The most reported patient determinant was *expectations from patients*, which was as a barrier to de-implementation in five studies [84–88]. This included patients’ opposition to de-implementation [84], patients’ request for tests and treatments [85, 86] and challenges overcoming patients’ preferences and values and community standards of care [88]. *Patient knowledge* was both a facilitator and a barrier to de-implementation. Patients’ being aware of the negative consequences of LVC facilitated de-implementation [89], whereas a lack of knowledge acted as a barrier [88].

#### Professional determinants

Professional determinants was the most common category. All studies described barriers to de-implementation. The most common subcategory was *professionals’ expectations, attitudes and behaviour* which consisted of clinicians resistance to change [84], fear of malpractice due to a “malpractice system” [85], lack of interest in  saving money [90] and fear of litigation [87, 88]. *Professionals’ knowledge* included one study that described a gap in professionals knowledge about their actual use of the LVC practice which meant that they overestimated to what extent they had reduced their use [86]. The subcategory *professionals’ memory*, had to do with clinicians’ forgetting to assess patients’ eligibility for a decreased use of a practice [86].

#### Outer context determinants

Outer context determinants were found to both facilitate and hinder de-implementation and comprised three subcategories.

Concerning *policy and political support*, having a clear rationale for change [91] facilitated de-implementations, and a weak political willingness [84] hindered de-implementation. One study found that a lack of physicians’ influence on policy concerning LVC practice had a positive influence on the intention to stop using the practice [90]. The same study found that *pressure from suppliers* to use the LVC practice had a positive relation to de-implementation. According to the authors, one possible explanation is that physicians may only perceive these to be barriers when they have the intention to stop using the LVC practice and feel hindered by these factors [90]. The subcategory *economy* encompassed one study showing that payment policies and performance measures that rewarded more services acted as barriers to de-implementation [85].

#### Inner context determinants

Inner context determinants were found to hinder as well as facilitate de-implementation and were related to four subcategories.

In the subcategory *setting characteristics*, one study found that academic medical centres performed better in relation to de-implementation, compared to for-profit and not-for-profit centres [92]. This was connected to the *organisational culture*, i.e. norms, values and incentives, of academic medical centres, which seemed to influence the behaviour not only of their regular employees, but also the freelancers working at the centres [92]. *Work/care process* was a potential barrier to de-implementation (*n* = 3) and included time pressure in general [86, 88], lack of time for shared decision-making [85] and lack of tools for shared decision-making [88]. *Interaction between professionals* (*n* = 1), i.e. the number of tests and treatments recommended by specialists, was a barrier to de-implementation [85].

#### Process determinants

Two studies identified process determinants related to the subcategory *de-implementation process.* A Delphi study proposed several determinants that may both hinder and facilitate de-implementation [91], with the most important being strength of executive leadership, strength of clinical leadership, quality of communication, clarity of specific aims and objectives, extent of cultural and behavioural change, attention to human aspects of process of change, quality of project management, availability of resources to support decision-making and implementation processes, quality of strategic planning and training of staff [91]. The other study identified complexity of the de-implementation process as a barrier when a specialised process or extensive patient education was required to reduce the LVC practice [86].

#### Evidence and LVC practice determinants

One study identified a lack of alternative practice to replace the LVC practice as a barrier to de-implementation [90]. Another study identified a lack of reliable and easily available information concerning current clinical practice and on safety, effectiveness and costs of health care interventions as a barrier for de-implementation [84].

## Discussion

This scoping review included a total of 101 studies of which the majority reported on determinants for the use of LVC (*n* = 92) and a limited number on determinants for de-implementation (*n* = 9). Our analysis resulted in six categories: patient determinants, professional determinants, inner context determinants, outer context determinants, process determinants and evidence and LVC practice determinants.

We used an inductive approach to develop the determinant categories although there are numerous frameworks to choose from within implementation science. However, while findings from implementation of evidence-based interventions might inform de-implementation research, it cannot be granted that knowledge is easily transferable between the two areas [8, 28]. Indeed, Davidson et al. [93] have called for a distinct de-implementation science that would “recognize and identify problem areas of low-value and wasteful practice, carry out rigorous scientific examinations of the factors that initiate and maintain such practices and then employ evidence-based interventions to extinguish these practices”.

The broad categories of determinants for the use and de-implementation of LVC largely overlap with implementation determinants as described in CFIR [12]. CFIR provides a relevant comparison because it is one of the most comprehensive and most widely applied frameworks [94]. CFIR is broadly similar to many other implementation determinant frameworks as there is considerable consensus as to what the main types of implementation determinants are [11]. CFIR encompasses five determinant domains (our categories in brackets): intervention characteristics (evidence and LVC practice determinants). inner setting (inner context determinants), outer setting (outer context determinants), characteristics of individuals (professional determinants) and process (process determinants [12]. In our review, patient determinants make up a unique category whereas in CFIR, patient needs and resources is a sub-domain that is part of the outer setting domain. The representation of patient determinants as a category of its own provided the most accurate representation of the data since patient determinants was a prominent determinant in the included studies. Furthermore, we identified two determinants that were more prominent with regard to the use of LVC and de-implementation of LVC than for implementation: *patient expectations* and *professionals’ fear of malpractice*.

Patient expectations are less commonly addressed in implementation frameworks (e.g. [12, 95, 96]). One possible reason for this difference may be that de-implementation involves reducing or stopping known practices that the patients are aware of and may previously have received. Implementation, on the other hand, often deals with implementing *new* practices that patients may not be aware of and, consequently, do not request. The influence of patients on the use of LVC should also be understood in light of factors influencing patients, e.g. advertisements and tests or treatments they are used to receiving. These factors can function as drivers as well as inhibitors of the use of LVC. For instance, extensive measures are undertaken to reduce antimicrobial resistance, including campaigns to raise awareness in the population [97]. By contrast, media attention to the positive effects of vitamin D has been suggested to have contributed to increased vitamin D lab tests [98], despite guidelines recommending against screening in the general population [99].

Professionals’ fear of malpractice, for instance, due to missing a diagnosis, or of becoming the target of litigation, was another determinant found in this review that is rarely identified as an implementation determinant. It may seem paradoxical that fear of malpractice was a driver of using LVC since providing LVC may be directly harmful to patients. However, a potential explanation is that some LVC practices, e.g. computed tomography scans of the head in patients with minor head injury [47], were considered relatively harmless, relative to the risk of missing a serious diagnosis. Fear of litigation is likely a more prominent determinant in countries that have a system in which such legal actions are fairly common.

Professionals’ attitudes and behaviours have been identified to influence both implementation [12] and de-implementation, but the reasons and mechanisms for how these influences operate may differ between the two processes, as has also been proposed by Norton and Chambers [100]. Reducing LVC may be difficult when health care professionals have developed familiarity and expertise with certain practices, to the extent that such practices become intertwined with their identity, position and status [101–103]. Helfrich et al. [104] suggest that attempts at de-implementation may be perceived by health care professionals as an attack or infringement on their personal prerogative, which can actually increase their commitment to the current LVC practice. There are also organisational and system-level influences that may inhibit efforts to reduce the use of LVC. The culture of teams, professions and organisations constitutes a stabilising influence by imposing norms, values, beliefs and assumptions that become internalised and tacitly held among members of the groups, thus functioning as “collective habits” shared among groups of people [105].

The process category was the least reported category in this review with only two broad subcategories (strategies and de-implementation process) of determinants identified. CFIR, on the other hand, outlines several different and more specific determinants (e.g. planning, engaging, executing, reflecting and evaluating). This makes it challenging to draw any firm conclusions concerning the extent to which process determinants for the use and de-implementation of LVC overlap with process determinants for implementation. In general, there is a paucity of research on de-implementation processes which may be due to difficulties of defining, measuring and evaluating this domain [106].

Consistent with CFIR, this review shows that determinants for use of LVC and de-implementation of LVC exist on all levels of the system, from the individual patient and professional to the work group, organisation and the wider health care system. Individual-level determinants were the most commonly identified determinants. A majority of the included studies were retrospective record reviews, which reported on associations between different determinants and the use of LVC. These studies were not designed to explore a range of potential determinants; rather, the focus was on the limited number of variables available in medical records, such as patient characteristics. This means that important determinants may have been neglected.

Despite the fairly large focus on individual-level determinants, the included studies show that individual professionals’ decision to provide or not provide LVC is affected by their context and the interactions with patients and with other professionals. This suggests that it is important to provide a work environment (e.g. peer-support) and work process (e.g. decision support) in which individual professionals are supported and have the prerequisites to resist LVC.

This underscores the importance of viewing health care systems in holistic terms, because the use of LVC likely depends on combinations of determinants. Furthermore, the multi-level determinants impacting the use of LVC and de-implementation of LVC point to the relevance of accounting for determinants at different levels when planning strategies to reduce the use of LVC. It is important to avoid an overly reductionist approach, studying the impact of different determinants in isolation of each other, since this neglects the fact that two or more seemingly unimportant determinants may create powerful effects if they are combined and potentially strong influences may combine to generate weak effects [5].

### Knowledge gaps and implications

The findings imply that existing implementation determinants are useful also for assessing determinants for the use and de-implementation of LVC. However, the results also show that adaptations may be required. We propose that greater consideration should be placed on how patient expectations and professionals’ fear of malpractice influence the use of LVC. These determinants should also be considered when planning strategies to de-implement LVC. The findings also imply that health care organisations must consider determinants at different levels when attempting to reduce LVC and that strategies solely targeting individual professionals may not be sufficient for reducing LVC.

Several areas in need of further research can be identified. First, the results point to a dearth of studies on determinants for the process of de-implementing LVC. Second, there is a need for studies that use study designs facilitating the investigation of the mechanisms underlying the determinants. Third, more research is needed to investigate how de-implementation determinants relate to existing determinant frameworks, including CFIR. There is especially a lack of studies that address determinants related to the process domain. Fourth, the existing literature illustrates a divergent effect of determinants. More research is needed to examine how determinants differ depending on the type of LVC practice. Fifth, the majority of the identified studies were conducted in the USA and the external validity of these studies is unclear. Thus, studies on de-implementation in other countries, including low- and middle-income countries, and health care systems (e.g., with different funding structures) are warranted.

### Methodological considerations

The applied methods had multiple strengths: the search was designed and performed with the university library and a structured process was applied [21]. Furthermore, screening and coding determinants were conducted by at least two authors and team discussions were applied to resolve queries. The data charting was conducted individually which implies a risk that discrepancies in data charting were missed. To mitigate this we performed a thorough testing of the data charting form and held team discussions about difficulties that occurred. Further, one of the authors performed a quality control of all the extracted data. It should be noted that although the coding and categorisation of determinants were conducted using an inductive approach the authors have considerable pre-existing knowledge concerning implementation determinant frameworks and implementation terminology which likely has influenced the understanding of the determinants as well as the labelling of categories.

The study has some limitations. The search for the review was conducted up to June 2018. This means that newer publications are not included in the results. The inconsistent terminology for LVC and de-implementation [25] and the range of clinical fields that the studies were conducted in entails a risk that studies may have been missed. Moreover, the review covers literature only in English and published in peer-reviewed sources. Also, the degree of evidence for the practice being of low value may differ between studies, as we relied on the referencing the studies made to clinical guidelines. Studies of different designs were included in the review to provide a broad overview of reported determinants for the use of LVC and de-implementation of LVC. Therefore, the review does not provide information about the strength of the associations of the identified determinants. Furthermore, the amount of information about the identified determinants in the included studies varied considerably which sometimes limited the information that could be reported in this review.

## Conclusion

This scoping review provides the first compilation of determinants for the use of LVC as well as de-implementation of LVC. The identified determinants largely overlap with existing implementation frameworks, although patient expectations and professionals’ fear of malpractice appear to be more prominent determinants for the use and de-implementation of LVC. Thus, existing implementation determinant frameworks may require adaptation to be transferable to de-implementation. Strategies for reducing the use of LVC should specifically consider determinants for the use of LVC and de-implementation of LVC.

## Supplementary Information

**Additional file 1.** Preferred Reporting Items for Systematic reviews and Meta-Analyses extension for Scoping Reviews (PRISMA-ScR) Checklist

**Additional file 2.** Documentation of search strategies

**Additional file 3.** Definitions of sub-categories and overview of individual codes included in each sub-category

**Additional file 4.** Table A. Information about included studies on determinants for the use of LVC. Table B. Information about included studies on determinants for the de-implementation of LVC

## Data Availability

The datasets used will be available from the corresponding author on reasonable request.

## Abbreviations

LVC: Low-value care
CFIR: Consolidated Framework for Implementation Research
