# Supplementary material for: Determinants for the use and de-implementation of low-value care in health care: a scoping review
Source: Implement Sci Commun. 2021 Feb 4;2:13. doi: 10.1186/s43058-021-00110-3 (PMC7860215; doi:10.1186/s43058-021-00110-3)
Supplement: Supplementary file 2 — Additional file 2. Documentation of search strategies [file 43058_2021_110_MOESM2_ESM.docx]

**Additional file 2.**

Documentation of search strategies

1. Medline

| Interface: Ovid  Date of Search: June 4, 2018  Number of hits: 2,903  Comment: In Ovid, two or more words are automatically searched as phrases; i.e. no quotation marks are needed | Field labels   - exp/ = exploded MeSH term - / = non exploded MeSH term - .ti,ab,kf. = title, abstract and author keywords - adjx = within x words, regardless of order - * = truncation of word for alternate endings |
| --- | --- |
| 1. exp Medical Overuse/  2. Inappropriate Prescribing/  3. Deprescriptions/  4. ((abandon* or contradict* or deadopt* or de-adopt* or disadopt* or dis-adopt* or decommission* or de-commission* or deimplement* or de-implement* or delist* or de-list* or disinvest* or dis-invest or deprescript* or deprescrib* or divest* or inapprop* or ineffective* or low-value or obsole* or outmoded or overuse or reallocate* or reassess* or re-assess* or refute* or refuting or re-invest* or medical revers* or supersed* or unlearn*) adj4 (care or clinic* or device* or drug or drugs or evidence* or health or healthcare or medical or medication* or prescrib* or procedur* or technolog* or therap* or treat*)).ti,ab,kf.  5. ((chang* or discontinu* or dis-continu* or decreas* or declin* or drop or reduc* or withdraw*) adj2 ("use" or practice) adj4 (care or clinic* or device* or drug or drugs or evidence* or health or healthcare or medical or medication* or prescrib* or procedur* or technolog* or therap* or treat*)).ti,ab,kf.  6. (choosing wisely or priority setting).ti,ab,kf.  7. (care or clinic* or device* or drug or drugs or evidence* or health or healthcare or medical or medication* or prescrib* or procedur* or technolog* or therap* or treat*).ti,ab,kf.  8. 6 and 7  9. 1 or 2 or 3 or 4 or 5 or 8  10. exp Evidence-Based Practice/  11. Program Evaluation/  12. Decision Making/  13. Practice Patterns, Physicians'/  14. Practice Patterns, Nurses'/  15. ((abandon* or contradict* or deadopt* or de-adopt* or disadopt* or dis-adopt* or decommission* or de-commission* or deimplement* or de-implement* or delist* or de-list* or disinvest* or dis-invest or discontinu* or dis-continu* or deprescipt* or deprescrib* or divest* or inapprop* or ineffective* or low-value or obsole* or outmoded or overuse or reallocate* or reassess* or re-assess* or refute* or refuting or re-invest* or medical revers* or supersed* or unlearn* or withdraw*) adj4 (factor* or barrier* or engag* or evidence-based or facilitat* or determinant* or predict* or model* or framework* or intervent* or policy or policies or practice pattern* or program* or strateg* or tool*)).ti,ab,kf.  16. or/10-15  17. 9 and 16   18. 17 not (animals not humans).sh.  19. limit 18 to english language | |

2. Embase

| Interface: embase.com  Date of Search: June 4, 2018  Number of hits: 4,733  Comment: Emtree is the controlled vocabulary in Embase | Field labels   - /exp = exploded Emtree term - /de = non exploded Emtree term - ti,ab = title and abstract - NEAR/x = within x words, regardless of order - * = truncation of word for alternate endings |
| --- | --- |
| #28 #25 NOT #26 AND [english]/lim  #27 #25 NOT #26  #26 'animals'/exp NOT 'humans'/exp  #25 #12 AND #24  #24 #13 OR #14 OR #15 OR #16 OR #17 OR #18 OR #19 OR #20 OR #21 OR #22 OR #23  #23 ((abandon* OR contradict* OR deadopt* OR 'de-adopt*' OR disadopt* OR 'dis-adopt*' OR decommission* OR 'de-commission*' OR deimplement* OR 'de-implement*' OR delist* OR 'de-list*' OR disinvest* OR 'dis-invest' OR discontinu* OR 'dis-continu*' OR deprescipt* OR deprescrib* OR divest* OR inapprop* OR ineffective* OR 'low-value' OR obsole* OR outmoded OR overuse OR reallocate* OR reassess* OR 're-assess*' OR refute* OR refuting OR 're-invest*' OR 'medical revers*' OR supersed* OR unlearn* OR withdraw*) NEAR/4 (factor* OR barrier* OR engag* OR 'evidence-based' OR facilitat* OR determinant* OR predict* OR model* OR framework* OR intervent* OR policy OR policies OR 'practice pattern*' OR program* OR strateg* OR tool*)):ti,ab,kw  #22 'evidence based emergency medicine'/exp  #21 'evidence based medicine'/de  #20 'evidence based nursing'/exp  #19 'evidence based dentistry'/exp  #18 'evidence based practice'/de  #17 'clinical decision making'/de  #16 'nursing practice'/exp  #15 'clinical practice'/exp  #14 'decision making'/de  #13 'program evaluation'/exp  #12 #1 OR #2 OR #4 OR #5 OR #8 OR #11  #11 #9 AND #10  #10 care:ti,ab,kw OR clinic*:ti,ab,kw OR device*:ti,ab,kw OR drug:ti,ab,kw OR drugs:ti,ab,kw OR evidence*:ti,ab,kw OR health:ti,ab,kw OR healthcare:ti,ab,kw OR medical:ti,ab,kw OR medication*:ti,ab,kw OR prescrib*:ti,ab,kw OR procedur*:ti,ab,kw OR technolog*:ti,ab,kw OR therap*:ti,ab,kw OR treat*:ti,ab,kw  #9 'choosing wisely':ti,ab,kw OR 'priority setting':ti,ab,kw  #8 #6 OR #7  #7 ((chang* OR discontinu* OR 'dis-continu*' OR decreas* OR declin* OR drop OR reduc* OR withdraw*) NEAR/2 use NEAR/4 (care OR clinic* OR device* OR drug OR drugs OR evidence* OR health OR healthcare OR medical OR medication* OR prescrib* OR procedur* OR technolog* OR therap* OR treat*)):ti,ab,kw  #6 ((chang* OR discontinu* OR 'dis-continu*' OR decreas* OR declin* OR drop OR reduc* OR withdraw*) NEAR/2 practice NEAR/4 (care OR clinic* OR device* OR drug OR drugs OR evidence* OR health OR healthcare OR medical OR medication* OR prescrib* OR procedur* OR technolog* OR therap* OR treat*)):ti,ab,kw  #5 ((abandon* OR contradict* OR deadopt* OR 'de-adopt*' OR disadopt* OR 'dis-adopt*' OR decommission* OR 'de-commission*' OR deimplement* OR 'de-implement*' OR delist* OR 'de-list*' OR disinvest* OR 'dis-invest' OR deprescript* OR deprescrib* OR divest* OR inapprop* OR ineffective* OR 'low-value' OR obsole* OR outmoded OR overuse OR reallocate* OR reassess* OR 're-assess*' OR refute* OR refuting OR 're-invest*' OR 'medical revers*' OR supersed* OR unlearn*) NEAR/4 (care OR clinic* OR device* OR drug OR drugs OR evidence* OR health OR healthcare OR medical OR medication* OR prescrib* OR procedur* OR technolog* OR therap* OR treat*)):ti,ab,kw  #4 'deprescription'/exp  #3 'inappropriate prescribing'/de  #2 'medication overuse'/exp  #1 'unnecessary procedure'/exp | |

3. Web of Science Core Collection

| Interface: Clarivate Analytics  Date of Search: June 4, 2018  Number of hits: 1,072 | Field labels   - TS/Topic = title, abstract, author keywords and Keywords Plus - NEAR/x = within x words, regardless of order - * = truncation of word for alternate endings |
| --- | --- |
| #1 TOPIC: (((abandon* OR contradict* OR deadopt* OR ”de-adopt*” OR disadopt* OR ”dis-adopt*” OR decommission* OR ”de-commission*” OR deimplement* OR ”de-implement*” OR delist* OR ”de-list*” OR disinvest* OR ”dis-invest” OR deprescript* OR deprescrib* OR divest* OR inapprop* OR ineffective* OR ”low-value” OR obsole* OR outmoded OR overuse OR reallocate* OR reassess* OR ”re-assess*” OR refute* OR refuting OR ”re-invest*” OR ”medical revers*” OR supersed* OR unlearn*) NEAR/3 (care OR clinic* OR device* OR drug OR drugs OR evidence* OR health OR healthcare OR medical OR medication* OR prescrib* OR procedur* OR technolog* OR therap* OR treat*)))  #2 (((chang* or discontinu* or ”dis-continu*” or decreas* or declin* or drop or reduc* or withdraw*) NEAR/1 ("use" or practice) NEAR/3 (care or clinic* or device* or drug or drugs or evidence* or health or healthcare or medical or medication* or prescrib* or procedur* or technolog* or therap* or treat*)))  #3 TS=("choosing wisely" or "priority setting") AND TS=(care or clinic* or device* or drug or drugs or evidence* or health or healthcare or medical or medication* or prescrib* or procedur* or technolog* or therap* or treat*)  #4 #3 OR #2 OR #1  #5 ((abandon* OR contradict* OR deadopt* OR ”de-adopt*” OR disadopt* OR ”dis-adopt*” OR decommission* OR ”de-commission*” OR deimplement* OR ”de-implement*” OR delist* OR ”de-list*” OR disinvest* OR ”dis-invest” OR discontinu* OR ”dis-continu*” OR deprescipt* OR deprescrib* OR divest* OR inapprop* OR ineffective* OR ”low-value” OR obsole* OR outmoded OR overuse OR reallocate* OR reassess* OR ”re-assess*” OR refute* OR refuting OR ”re-invest*” OR ”medical revers*” OR supersed* OR unlearn* OR withdraw*) NEAR/3 (factor* OR barrier* OR engag* OR ”evidence-based” OR facilitat* OR determinant* OR predict* OR model* OR framework* OR intervent* OR policy OR policies OR ”practice pattern*” OR program* OR strateg* OR tool*))  #6 #5 AND #4 Refined by: LANGUAGES: ( ENGLISH ) | |

4. Cinahl

| Interface: Ebsco  Date of Search: June 4, 2018  Number of hits: 934 | Field labels   - MH+ = exploded Cinahl Heading - MH = non exploded Cinahl Heading - TI = title - AB = abstract - Nx = within x words, regardless of order - * = truncation of word for alternate endings |
| --- | --- |
| Limiters - English Language  S16 S8 AND S15  S15 S9 OR S10 OR S11 OR S12 OR S13 OR S14  S14 TI ( (abandon* OR contradict* OR deadopt* OR ”de-adopt*” OR disadopt* OR ”dis-adopt*” OR decommission* OR ”de-commission*” OR deimplement* OR ”de-implement*” OR delist* OR ”de-list*” OR disinvest* OR ”dis-invest” OR discontinu* OR ”dis-continu*” OR deprescipt* OR deprescrib* OR divest* OR inapprop* OR ineffective* OR ”low-value” OR obsole* OR outmoded OR overuse OR reallocate* OR reassess* OR ”re-assess*” OR refute* OR refuting OR ”re-invest*” OR ”medical revers*” OR supersed* OR unlearn* OR withdraw*) N3 (factor* OR barrier* OR engag* OR ”evidence-based” OR facilitat* OR determinant* OR predict* OR model* OR framework* OR intervent* OR policy OR policies OR ”practice pattern*” OR program* OR strateg* OR tool*) ) OR AB ( (abandon* OR contradict* OR deadopt* OR ”de-adopt*” OR disadopt* OR ”dis-adopt*” OR decommission* OR ”de-commission*” OR deimplement* OR ”de-implement*” OR delist* OR ”de-list*” OR disinvest* OR ”dis-invest” OR discontinu* OR ”dis-continu*” OR deprescipt* OR deprescrib* OR divest* OR inapprop* OR ineffective* OR ”low-value” OR obsole* OR outmoded OR overuse OR reallocate* OR reassess* OR ”re-assess*” OR refute* OR refuting OR ”re-invest*” OR ”medical revers*” OR supersed* OR unlearn* OR withdraw*) N3 (factor* OR barrier* OR engag* OR ”evidence-based” OR facilitat* OR determinant* OR predict* OR model* OR framework* OR intervent* OR policy OR policies OR ”practice pattern*” OR program* OR strateg* OR tool*) )  S13 (MH "Practice Patterns")  S12 (MH "Decision Making, Clinical")  S11 (MH "Decision Making")  S10 (MH "Program Evaluation")  S9 (MH "Professional Practice, Evidence-Based+")  S8 S1 OR S2 OR S3 OR S4 OR S7  S7 S5 AND S6  S6 TI ( care or clinic* or device* or drug or drugs or evidence* or health or healthcare or medical or medication* or prescrib* or procedur* or technolog* or therap* or treat* ) OR AB ( care or clinic* or device* or drug or drugs or evidence* or health or healthcare or medical or medication* or prescrib* or procedur* or technolog* or therap* or treat* )  S5 TI ( "choosing wisely" or "priority setting" ) OR AB ( "choosing wisely" or "priority setting" )  S4 TI ( (((chang* or discontinu* or ”dis-continu*” or decreas* or declin* or drop or reduc* or withdraw*) N1 (use or practice)) N3 (care or clinic* or device* or drug or drugs or evidence* or health or healthcare or medical or medication* or prescrib* or procedur* or technolog* or therap* or treat*)) ) OR AB ( (((chang* or discontinu* or ”dis-continu*” or decreas* or declin* or drop or reduc* or withdraw*) N1 (use or practice)) N3 (care or clinic* or device* or drug or drugs or evidence* or health or healthcare or medical or medication* or prescrib* or procedur* or technolog* or therap* or treat*)) )  S3 TI ( (abandon* OR contradict* OR deadopt* OR ”de-adopt*” OR disadopt* OR ”dis-adopt*” OR decommission* OR ”de-commission*” OR deimplement* OR ”de-implement*” OR delist* OR ”de-list*” OR disinvest* OR ”dis-invest” OR deprescript* OR deprescrib* OR divest* OR inapprop* OR ineffective* OR ”low-value” OR obsole* OR outmoded OR overuse OR reallocate* OR reassess* OR ”re-assess*” OR refute* OR refuting OR ”re-invest*” OR ”medical revers*” OR supersed* OR unlearn*) N3 (care OR clinic* OR device* OR drug OR drugs OR evidence* OR health OR healthcare OR medical OR medication* OR prescrib* OR procedur* OR technolog* OR therap* OR treat*) ) OR AB ( (abandon* OR contradict* OR deadopt* OR ”de-adopt*” OR disadopt* OR ”dis-adopt*” OR decommission* OR ”de-commission*” OR deimplement* OR ”de-implement*” OR delist* OR ”de-list*” OR disinvest* OR ”dis-invest” OR deprescript* OR deprescrib* OR divest* OR inapprop* OR ineffective* OR ”low-value” OR obsole* OR outmoded OR overuse OR reallocate* OR reassess* OR ”re-assess*” OR refute* OR refuting OR ”re-invest*” OR ”medical revers*” OR supersed* OR unlearn*) N3 (care OR clinic* OR device* OR drug OR drugs OR evidence* OR health OR healthcare OR medical OR medication* OR prescrib* OR procedur* OR technolog* OR therap* OR treat*) )  S2 (MH "Inappropriate Prescribing")  S1 (MH "Unnecessary Procedures") | |
