# Supplementary material for: Determinants for the use and de-implementation of low-value care in health care: a scoping review
Source: Implement Sci Commun. 2021 Feb 4;2:13. doi: 10.1186/s43058-021-00110-3 (PMC7860215; doi:10.1186/s43058-021-00110-3)
Supplement: Supplementary file 3 — Additional file 3. Definitions of sub-categories and overview of individual codes included in each sub-category [file 43058_2021_110_MOESM3_ESM.docx]

**Additional file 3.**

Definitions of sub-categories and overview of individual codes included in each sub-category

| **Determinants** | **Definitions** | **Individual codes in the sub-categories** |
| --- | --- | --- |
| **Patient determinants** | | |
| Patient characteristics | Characteristics of the individual patient | Age; gender; race; high health care consumer; having a relative that is a physician; income; marital status; socioeconomic status; education; smoker |
| Patient health condition | Characteristics of patients’ health condition | Comorbidity; severity of illness; number of drugs; type of diagnosis; type of health condition; type of bacteria; length of disease; length of stay in hospital; psychiatric disorder; depression; stage of disease; health care history |
| Patient expectations | Patients’ expectations, demands and requests for LVC | Patient refusal; patient request; patient demand; patient expectations; patient pressure; patient desire; patient preferences |
| Patient knowledge | Patients’ knowledge about LVC and harms of LVC | Patient knowledge about LVC; patient knowledge about harms of LVC |
| Expectations from relatives | Expectations and demands from patients’ family members and relatives | Family member expectation; demands from relatives |
| **Professional determinants** | | |
| Professional characteristics | Characteristics of the individual health care professional | Age; gender; race; type of specialisation; years in profession; lack of experience; years of training; length of experience; place of training; generally risk averse; lack of consultation skills; personality; medical school affiliation |
| Professional knowledge of LVC | Healthcare professionals’ knowledge about LVC and its potential consequences and their knowledge about how to manage LVC requests | Lack of cost awareness; knowledge about LVC guidelines; difficult to stay updated about guidelines; misconceptions about LVC; awareness of risk with LVC; training in how to recommend against LVC |
| Professionals’ expectations, attitudes and behaviours | Health care professionals’ expectations, attitudes and behaviours influencing the use of LVC and de-implementation of LVC | Fear of malpractice; desire to meet patient request; fear of litigation; fear of not meeting supervisor expectations; habit performing LVC; reassure patient; self-efficacy to discuss LVC with patients; self-efficacy to deny patients LVC; perception of guidelines; motivation; perception of consequences of LVC; individual cost-consciousness |
| Professionals’ memory | Healthcare professionals forgetting to look for eligibility/indication for a practice | Staff forgetting |
| **Outer context determinants** | | |
| Location | The geographical area where the use of LVC is being investigated | Region; urban area; rural area; suburban area; metropolitan area |
| Economy | Financing of health care and financial incentives influencing the use of LVC and de-implementation of LVC | Insurance type; economic incentives for LVC; reimbursement/funding models; patients not paying for care; lack of money |
| Outer context characteristics | Characteristics of the social, political and geographical context of health care | Health care consumption in the region; ratio of specialist to primary care physician; proportion of residents with poorer health; specialist density; population with low risk for disease; high physician concentration; low prevalence of the LVC; high physician concentration; socio-economy |
| Patient volume | The volume of patients in specific health care settings | Patient volume; patient visits/physician |
| Policy and political support | Policies and political support influencing the use of LVC and de-implementation of LVC | Policy for reducing LVC; lack of political support |
| Marketing | Market incentives and marketing influencing the use of LVC | Advertising to consumers; promotion of screening; market incentives |
| Time | Year of diagnosis or treatment | Year of diagnosis; year of treatment |
| Pressure from suppliers | Pressure from suppliers to use LVC practices | Pressure from suppliers to use the LVC practice |
| **Inner context determinants** | | |
| Setting characteristics | The characteristics of the health care setting where the use of LVC is being investigated | Type of setting (e.g. family practice; ambulatory care); size of the setting; ownership of practice |
| Work/care process | Work or care processes influencing the use of LVC and de-implementation of LVC | Number of handoffs; continuity of care; time pressure; perceived inaccessibility/lack of usefulness of decision support; several care providers; individual rather than clinic based decision making; efficiency and throughput; complex prescribing environment; automatized processes; streamlined referrals; teamwork |
| Staff composition | The composition of staff at the specific health care setting | Inadequate staffing; solo practise |
| Organisational structures related to the LVC | Structures in the organisation that provide incentives for or facilitate use of LVC | Ease of ordering repeating labs;  Organisational structures that provide incentives for LVC;  Ownership of equipment;  High ordering practice compared to low ordering practice |
| Interaction between professionals | Interaction and communication between health care professionals influencing the use of LVC | Lack of communication between professionals; expectations from specialist; requested by other professional; specialist referral |
| Culture | Organisational culture influencing the use of LVC and de-implementation of LVC | Hierarchical culture; lack of debate; screening culture; lack of cost consciousness; organizational norms |
| Patient-provider communication/interaction | Communication or interaction between health care providers and patients influencing the use of LVC | Patient and provider communication; patient-provider relationship |
| **Process determinants** | | |
| Strategies | Strategies for managing LVC | Audit and feedback; patient education; provider education; decision support; processes and routines for managing LVC |
| De-implementation process | Processes for de-implementing LVC | Complexity of de-implementation process; pace; quality of planning; quality of project management; training |
| **Evidence and LVC practice determinants** | | |
| Evidence | Evidence and guidelines related to the LVC practice and alternative practices | Conflicting guidelines; guidelines not applicable; lack of access to guidelines/evidence; perceived trustworthiness of evidence; lack of guidelines for alternative interventions; release of new guideline; demonstrable benefits and clarity of rationale for change |
| Characteristics of the LVC | Characteristics of the LVC practice influencing its use | LVC practice easy to distribute; lack of negative consequences of the LVC practice |
| Negative consequences of reducing LVC for the professional | Negative consequences of reducing LVC for the health care professionals | Fear of losing expertise |
| Characteristics of alternative practice | Characteristics of alternative practice to substitute the LVC practice | Lack of alternative practice; lack of access to alternative practice (e.g. due to high demand) |
