# Supplementary material for: Determinants for the use and de-implementation of low-value care in health care: a scoping review
Source: Implement Sci Commun. 2021 Feb 4;2:13. doi: 10.1186/s43058-021-00110-3 (PMC7860215; doi:10.1186/s43058-021-00110-3)
Supplement: Supplementary file 4 — Additional file 4. Table A. Information about included studies on determinants for the use of LVC. Table B. Information about included studies on determinants for the de-implementation of LVC [file 43058_2021_110_MOESM4_ESM.docx]

**Additional File 4.**

**Table A.** Information about included studies on determinants for the use of LVC

| **Reference** | **Country** | **Setting** | **Design** | **Methods** | **Number and type of respondents** | **Low-value care practice** | **Guideline/**  **recommendation** | **Determinants** |
| --- | --- | --- | --- | --- | --- | --- | --- | --- |
| Sharp et al 2015[[1](#_ENREF_1)] | USA | Primary care, hospital | Quantitative | Record review | 152 774 encounters | Antibiotics and a computed tomography (CT) scan of the face, head, or sinuses for acute sinusitis | Choosing Wisely | Setting |
| Scherer et al 2013[[2](#_ENREF_2)] | USA | Primary care | Quantitative | Survey | 175 parents of children presenting to a primary care paediatrics clinic | Acid-reducing medication for gastroesophageal reflux disease (GERD) in infants | North American  infant GERD clinical care guidelines | Disease label |
| Weinberg et al 2016[[3](#_ENREF_3)] | USA | Hospital | Quantitative | Record review | 124 462 patient encounters | Robotic radical nephrectomy | EAU guidelines on robotic and single-site surgery in urology. | Patient volume, Setting |
| Singh et al. 2016[[4](#_ENREF_4)] | USA | Community hospital | Quantitative | Survey | 588 adult patients | Acid Suppressive Therapy | Internal guideline based on the ASHP Therapeutic Guidelines on Stress Ulcer Prophylaxis, | Patient characteristics, Work/care process, |
| Schubert et al 2013[[5](#_ENREF_5)] | Germany | Not specified | Quantitative | Record review | 73 665 insurants | Potentially inappropriate medication | PRISCUS-PIM | Patient characteristics, Health condition |
| Ladapo et al 2014[[6](#_ENREF_6)] | USA | Office-based  physicians and hospital-based outpatient clinics | Quantitative | Survey | 8 645 encounters with adults without a diagnosis of coronary heart disease, | Cardiac stress test | American College of Cardiology Foundation Appropriate Use Criteria  Task Force, Choosing Wisely. | Patient characteristics, Professional characteristics, Economy |
| Lam et al 2014[[7](#_ENREF_7)] | Canada | Community family medicine | Quantitative | Survey | 58 Family physicians | Inappropriate antibiotic use for urinary tract infections | Anti-infective Guidelines for Community Acquired Infections in Ontario | Patient expectations, Work/care process |
| Rasu et al 2014[[8](#_ENREF_8)] | Bangladesh | Village healthcare | Quantitative | Survey | 77 Village Doctors, 2 587 patient prescriptions | Inappropriate use of medications | WHO Integrated management of childhood  illness (IMCI) | Health condition, Professional characteristics |
| Hines et al 2014[[9](#_ENREF_9)] | USA | Hospitals | Quantitative | Survey | 279 Physicians (generalists and specialists) | Low-value care diagnostic tests | List of 37 low-value diagnostic tests compiled by the American College of Physicians | Professional characteristics, Patient expectations Setting, Economy, Professional knowledge of LVC, Professional, expectations, attitudes and behaviours, Marketing |
| Sedrak et al 2016[[10](#_ENREF_10)] | USA | Hospital | Mixed methods | Survey | 116 Internal medicine and general surgery residents | Inpatient ordering of unnecessary and potentially harmful laboratory test | Choosing Wisely | Professional characteristics, Professional knowledge of LVC, Professional, expectations, attitudes and behaviours, Culture |
| Mafi et al 2017[[11](#_ENREF_11)] | USA | Outpatient offices | Quantitative | Survey | 31 162 outpatients | the use of antibiotics and/or use of radiograph for upper respiratory tract infection, CT, MRI or radiograph for back pain, MRI for headache | Choosing Wisely | Setting, Work/care process, |
| Nuytten et al, 2017[[12](#_ENREF_12)] | USA | Neonatal intensive care | Quantitative | Survey | Data collected pertaining to 3 917 infants | Use of postnatal corticosteroid in preterm births below 30 weeks | Academy of Paediatrics and Canadian Paedtriatic Society guidelines. | Patient characteristics, Health condition, Policy |
| Wang et al 2017[[13](#_ENREF_13)] | USA | Not specified | Quantitative | Survey | 167 709 observations in children | Intranasal steroids to treat otitis media with effusion (OME) | 2016 multidisciplinary OME clinical practice guideline | Setting, Health condition |
| Kerns et al 2018[[14](#_ENREF_14)] | USA | Primary care | Qualitative | Interviews | 26 Primary care Physicians | Medications for behavioural and psychological symptoms of dementia (BPSD | National Initiative to Improve Behavioural Health and Reduce the Use of Antipsychotic Medications in Nursing Home Residents | Setting, work/care process  Professional, expectations, attitudes and behaviours, Evidence, Characteristics of alternative practice, Characteristics of the LVC,  Strategies, Professional s knowledge of LVC |
| Redwood et al 2018[[15](#_ENREF_15)] | USA | Hospital, academic center | Qualitative | Focus groups | 11 nurses | Unnecessary urine culture ordering and collecting practices | Recommendation by the Infectious Disease Society of America | Professional knowledge of LVC, Work/care process, Interaction between professionals |
| Schmidt et al 2018[[16](#_ENREF_16)] | USA | Ambulatory care | Quantitative | Record review | 898 ambulatory care practitioners, 281 315 patients | Inappropriate antimicrobial prescribing | American College of Physicians and the Centers for Disease Control and Prevention | Patient characteristics, Setting , Professional characteristics, Economy, Location |
| Hong et al 2017[[17](#_ENREF_17)] | USA | Not specified | Quantitative | Record review | 100 977 clinicians  878 720 patients | Low-value back imaging, Low-value head image | Choosing Wisely | Organisational structures related to the LVC, Professional, expectations, attitudes and behaviour |
| Irfan et al 2015[[18](#_ENREF_18)] | Canada | Hospital | Quantitative | Record Review | 160 patients with asymptomatic bacteriuria | Antimicrobials for asymptomatic bacteriuria | Infectious Diseases Society of America guidelines for the diagnosis and treatment of asymptomatic bacteriuria in adult | Patient characteristics, Health condition |
| Jiron et al 2016[[19](#_ENREF_19)] | USA | Aged care | Quantitative | Record Review | 38 250 patients | Potentially inappropriate medication | Beers criteria | Patient characteristics, Location, Health condition, Work/care process |
| Kachalia et al 2015[[20](#_ENREF_20)] | USA | Hospital | Quantitative | Survey (vignettes) | 1 020 members of society of hospital medicine | Pre-operative testing, syncope testing | American Heart Association Councils on Clinical Cardiology, Cardiovascular Nursing, Cardiovascular Disease in the Young, and Stroke | Patient characteristics Patient expectations, Professional, expectations, attitudes and behaviours, Evidence, Setting |
| Kachru et al 2015[[21](#_ENREF_21)] | USA | Aged care | Quantitative | Survey | 7 352 patients | Potentially inappropriate anticholinergic medications | Beers Criteria | Patient characteristics, Location |
| Kepka et al 2014[[22](#_ENREF_22)] | USA | Not specified | Quantitative | Survey | 9 494 patients | Pap tests | American Cancer Society, American Society for Colposcopy and Cervical Pathology, and American Society for Clinical Pathology screening guidelines for the prevention and early detection of cervical cancer | Patient characteristics, Economy |
| Klang et al 2017[[23](#_ENREF_23)] | Israel | Emergency care | Quantitative | Record Review | 955 patients | CT examinations for minor head trauma | A top-five list for emergency medicine: a pilot project to improve the value of emergency care and CCHR | Professional characteristics |
| Kondo et al 2014[[24](#_ENREF_24)] | Japan | Dialysis facilities | Quantitative | Survey | 1 367 patients over 65 undergoing regular dialysis | PIM according to Beers criteria | Beers criteria | Health condition, Work/care process, Setting |
| Kruse et al 2015[[25](#_ENREF_25)] | USA | Primary care | Quantitative | Record review | 1 429 patients | Overuse of colonoscopy for colorectal cancer screening and surveillance | Choosing Wisely | Interaction between professionals |
| Kwon et al 2016[[26](#_ENREF_26)] | USA | Hospital | Quantitative | Record review | 2 081 patients | Thrombophilia screening | British Committee for Standards in Haematology. Clinical guidelines for testing for heritable thrombophilia | Professional characteristics |
| Lalude et al 2014[[27](#_ENREF_27)] | USA | University medical center | Quantitative | Record review | 420 patients | Inappropriate use of single photon emission computed  tomography myocardial perfusion imaging (SPECT MPI) | AUC by American College of Cardiology and the American Society of Nuclear Cardiology | Patient characteristics |
| Lim et al 2017[[28](#_ENREF_28)] | USA | Not specified | Quantitative | Survey | 21 290 patients | Inappropriate use of antidepressants | U.S. Food and Drug Administration | Patient characteristics, Economy |
| Lin et al 2017[[29](#_ENREF_29)] | USA | Emergency care | Quantitative | Survey | 765 emergency physicians | Choosing Wisely recommendations (Head CT (trauma), IV fluids (peds),  Foley catheter.  Back pain imaging,  Chest CT (neg D-dimer),  Head CT (syncope),  Palliative care,  Antibiotics for abscess,  CT for renal colic,  Antibiotics for sinusitis) | Choosing Wisely | Patient expectations, Professional knowledge of LVC, Professional, expectations, attitudes and behaviours, Expectations from relatives |
| Lipitz-Snyderman et al 2016[[30](#_ENREF_30)] | USA | Not specified | Quantitative | Record review | 11 563 physicians,  194 050 patients with cancer | Imaging for staging and surveillance in low-risk disease, intensity-modulated radiation therapy (IMRT) after breast-conserving surgery and extended fractionation schemes for palliation of bone metastases. | Choosing Wisely | Professional, expectations, attitudes and behaviours, Organisational structures related to the LVC |
| Lund et al 2013[[31](#_ENREF_31)] | USA | Primary care | Quantitative | Record review | 1 549 824 patients | Potentially inappropriate medications | Zhan criteria and Fick criteria | Location |
| Mahal et al 2017[[32](#_ENREF_32)] | USA | Not specified | Quantitative | Record review | 190 050 patients undergoing hysterectomy | Oophorectomy during hysterectomy for benign disease | American College of Obstetricians and Gynaecologists guideline | Patient characteristics, Economy, Location |
| Makarov et al 2015 [[33](#_ENREF_33)] | USA | Not specified | Quantitative | Record review | 42 196 patients with prostate cancer | Prostate cancer imaging | Choosing Wisely | Patient characteristics, Setting, Economy, Location, Time, Patient volume |
| Makarov et al 2015[[34](#_ENREF_34)] | USA | Not specified | Quantitative | Record review | 9 219 men with prostate cancer and 30 398 women with breast cancer | Inappropriate prostate and breast cancer imaging | Choosing Wisely | Patient characteristics, Location, Time |
| Malo et al 2016[[35](#_ENREF_35)] | Spain | Primary care and hospitals | Quantitative | Record review | 48 255 patients with acute bronchitis | Inappropriate antibiotics for acute bronchitis | Respiratory Tract Infections–Antibiotic Prescribing Guideline of the, NICE, Nationwide Guideline on Antimicrobial Therapy in the Area of Aljarafe | Patient characteristics |
| Marchisio et al 2014[[36](#_ENREF_36)] | Italy | Not specified | Quantitative | Survey | 1 270 paediatricians and 852 otolaryngologists | Inappropriate antibiotics for acute otitis media | AOM treatment guidelines by the American Academy of Paediatrics | Professional characteristics |
| Means et al 2014[[37](#_ENREF_37)] | Uganda | Health centers | Quantitative | Survey | 45 591 patients with malaria | Inappropriate antibiotics for patients with malaria | WHO Integrated Management of Childhood Illness | Professional characteristics, Location, Characteristics of alternative practice, Health condition, Outer context characteristics |
| Moralidis et al 2013[[38](#_ENREF_38)] | Greece | Hospital | Qualitative | Interviews, Record review | 3 032 patients | Inappropriate myocardial perfusion imaging | MPI appropriate use criteria (AUC) | Professional characteristics, Patient characteristics |
| Munoz-Plaza et al 2016[[39](#_ENREF_39)] | USA | Primary care and urgent care | Qualitative | Interviews | 9 primary care and urgent care physicians | Non-recommended antibiotics for treatment of acute sinusitis. | Choosing Wisely | Patient characteristics, Professionals knowledge of LVC, Evidence, Patient-provider communication/interaction, Strategies |
| Pickles et al 2016[[40](#_ENREF_40)] | UK, Australia | Primary care | Qualitative | Interviews | 69 General practitioners | PSA testing for asymptomatic men | Australian Health Technology Advisory Committee,  Australian Cancer Society. Prostate cancer screening: guidelines for health professionals | Patient expectations, Setting, Economy, Work/care process, Professional, expectations, attitudes and behaviours, Interaction between professionals, Marketing, Culture, Evidence,  Professional knowledge about LVC, Policy |
| Rosenthal et al 2018[[41](#_ENREF_41)] | USA | Not specified | Quantitative | Insurance data review | 45 Million adult patients with an employer sponsored insurance | Imaging early in the course of new back pain, vitamin D deficiency screening for low-risk patients,  prescription opioids for migraine, cardiac testing in low-risk patients, short-interval bone  densitometry (DEXA), and preoperative cardiac testing in low-risk patients ahead of low risk (non-cardiac) surgery | Choosing Wisely | Economy, Work/care process |
| Ryskina et al 2017[[42](#_ENREF_42)] | USA | Not specified | Quantitative | Survey | 902 internal medicine physicians | X-ray and MRI imaging for acute low back pain; antibiotics for  sinusitis; breast, prostate, and colon cancer screening for patients with life expectancy of less  than 10 years; ECG testing for asymptomatic patients; and CT scan as the initial test for low  risk patients with possible venous thromboembolism (VTE). | Choosing Wisely | Professional attitudes and behaviour, Professional knowledge of LVC |
| Sawan et al 2017[[43](#_ENREF_43)] | Australia | Aged care | Qualitative | Interviews | 40 professionals | Use of psychotropic medicines for elderly | The Omnibus Budget Reconciliation Act,  and Australian Pharmaceutical Advisory Council guidelines | Professional characteristics, Staff composition, Strategies, Work/care process |
| Sawan et al 2016[[44](#_ENREF_44)] | Australia | Aged care | Qualitative | Interviews | 40 professionals | Use of psychotropic medicines for elderly | The Omnibus Budget Reconciliation Act,  and Australian Pharmaceutical Advisory Council guidelines | Strategies |
| Silverman et al 2017[[45](#_ENREF_45)] | Canada | Primary care | Quantitative | Record review | 8 990 Primary care physicians,  185 014 patients | Inappropriate prescribing for nonbacterial acute upper respiratory tract infection | Appropriate antibiotic use for acute respiratory tract infection in adults: advice for high-value care from the American College of Physicians and the Centers for Disease Control and Prevention. | Professional characteristics, patient volume |
| Simos et al 2015[[46](#_ENREF_46)] | Canada | Not specified | Quantitative | Survey | 173 Medical oncologists and breast cancer surgeons | Routine radiological evaluation for metastases in women with early stage breast cancer | Alberta Health Services; American Society of Clinical Oncology; British Columbia Cancer Agency; Cancer Care Ontario; European Society for Medical Oncology; National Comprehensive Cancer Network | Health condition |
| Tavakoli et al 2018[[47](#_ENREF_47)] | Canada | Not specified | Quantitative | Retrospective review of administrative health data | 343 520 asthma patients | Inappropriate use of short-acting beta agonist in asthma treatment | Canadian asthma consensus  conference summary of recommendations | Patient characteristics, Health condition, Work/care process |
| Undela et al 2014[[48](#_ENREF_48)] | USA | Hospital | Quantitative | Record review | 502 Patients over 60 years old | Potentially inappropriate medication | Beers criteria | Patient characteristics, Health condition, Work/care process |
| Winchester et al 2013[[49](#_ENREF_49)] | USA | Hospital | Quantitative | Record review | 582 veterans who underwent myocardial perfusion imaging | Inappropriate use of myocardial perfusion imaging | ACCF/ASNC/ACR/AHA/ASE/SCCT/ SCMR/SNM 2009 appropriate use criteria for cardiac radionuclide imaging | Health condition |
| Yaeger et al 2015[[50](#_ENREF_50)] | USA | Academic clinics | Quantitative | Record review | 20 581 children with upper respiratory tract infections | Inappropriate antibiotic prescription for upper respiratory tract infections | HEDIS 2011 measure, “Appropriate treatment for children with upper respiratory infection” | Patient characteristics, Professional characteristics, |
| Chang et al 2014[[51](#_ENREF_51)] | Taiwan | Aged care | Quantitative | Record review | 25 187 patient encounters | Potentially Inappropriate Medication | Beers criteria, PIM-Taiwan criteria, and the PRISCUS | Patient characteristics, Professional characteristics, Health condition |
| Colla et al 2015[[52](#_ENREF_52)] | USA | Not specified | Quantitative | Record review | 100% Medicare administrative claims data (2006–  2011) for all medicare beneficiaries over the age of 65 (number not specified) | 11 Choosing Wisely recommendations | Choosing Wisely | Patient characteristics, Outer context characteristics |
| Cuba Fuentes et al 2016[[53](#_ENREF_53)] | Peru | Primary care | Quantitative | Survey | 40 Family doctors | Choosing Wisely recommendations | Choosing Wisely | Patient expectations, Professional characteristics, Policy and political support, Professional, expectations, attitudes and behaviours |
| Botica et al 2013[[54](#_ENREF_54)] | Croatia | Primary care | Quantitative | General practitioners recorded data during patient visits | 51 General practitioners, 699 patients | Unnecessary prescription of antibiotics for tonsillopharyngitis | Ministry of Health and Social Welfare Republic of Croatia. MATRA Project control of antimicrobial resistance in Croatia. Matra Project MAT 05/HR/9/2. | Day of the week (Wednesdays) |
| Faustino et al 2013[[55](#_ENREF_55)] | Brazil | Hospital | Quantitative | Record review | 1 270 patients ≥ 60 years | Potentially inappropriate medicines | The 2003 version of the Beers criteria | Patient characteristics |
| Colla et al 2015[[56](#_ENREF_56)] | USA | Not specified | Quantitative | Record review | Medicare beneficiaries, 8.2 million low-risk personyears and 10.1 million high-risk personyears | Non-indicated cardiac testing in low-risk patients | Choosing Wisely | Outer context characteristics |
| Cope et al 2016[[57](#_ENREF_57)] | UK | Dentistry | Quantitative | Survey, record review | 590 clinical encounters with 42 General dental practitioners | Non-indicated antibiotic prescribing | Clinical guidelines by the Scottish Dental Clinical Effectiveness Programme (SDCEP) and Faculty of General Dental Practice (UK) | Patient expectations, Work/care process |
| Grigoryan et al 2017[[58](#_ENREF_58)] | USA | Primary care | Quantitative | Record review | 3134 patients with acute bronchitis diagnosis | Antibiotic treatment for uncomplicated acute bronchitis | Guidelines from the American College of Physicians and American Academy of Family Physicians | Patient characteristics |
| Gieniusz et al 2018[[59](#_ENREF_59)] | USA | Hospital | Quantitative | Survey | 168 attending physicians, residents, fellows | Percutaneous feeding tubes in patients with advanced dementia | American Geriatrics Society (AGS), Choosing Wisely | Patient characteristics, Patient expectations, Setting, Professional, expectations, attitudes and behaviours, Expectations from relatives |
| Dempsey et al 2014[[60](#_ENREF_60)] | USA | Primary care | Qualitative | Interviews | 12 medical doctors and 1 nurse practitioner | Prescribing antibiotics for cough/acute bronchitis | ACCP evidence-based clinical practice guidelines | Patient expectations, Economy, Professional knowledge of LVC, Work/care process, Professional, expectations, attitudes and behaviours |
| Michel-Lepage et al 2013[[61](#_ENREF_61)] | France | Primary care | Quantitative | Survey | 1 093 General practitioners | Antibiotics for tonsillitis in  children if Rapid Antigen Diagnostic Tests is not used | National guidelines | Professional characteristics, Location |
| Alber et al 2017[[62](#_ENREF_62)] | Germany | Primary care | Qualitative | Interviews | 13 General practitioners | LVC in general | Choosing Wisely | Patient expectations, Professional characteristics, Economy, Professional, expectations, attitudes and behaviours, Marketing, Culture, Organisational structures related to the LVC |
| Amos et al 2014[[63](#_ENREF_63)] | Italy | Not specified | Quantitative | Record review | 865 354 older adults | Potentially inappropriate medications | The 2007 Maio criteria list | Patient characteristics, Professional characteristics, Patient volume, Staff composition |
| Barlam et al 2015[[64](#_ENREF_64)] | USA | Hospital associated ambulatory practices | Quantitative | Record review | 79 physicians, 4 942 patients visits | Inappropriate antibiotic prescribing for acute respiratory tract infections |  | Patient characteristics, Setting |
| Barnett et al 2017[[65](#_ENREF_65)] | USA | Office–based physicians’  practices, outpatient departments | Quantitative | Survey | 193 062 office visits | 9 previously identified LVC practices. Antibiotics for URIs, CT scan for sinusitis,  Screening EKG in GME ,  Screening CBC in GME,  Screening UA in GME,  Narcotics for back/neck pain,  CT/MRI for back/neck pain,  Narcotics for headache,  CT/MRI for headache | Various guidelines referred to | Economy |
| Bell et al 2013[[66](#_ENREF_66)] | Norway | Primary care | Qualitative | Focus groups | 13 General Practitioners | Fall-risk-increasing drugs | Norwegian General Practice Criteria | Patient expectations, Work/care process, Evidence, Other (patient already using the LVC practice) |
| Beuscart et al 2017[[67](#_ENREF_67)] | France | Not specified | Quantitative | Record review | 207 979 people aged 75 years and over liv | Potentially inappropriate medications | Potentially inappropriate medications in the elderly: a French consensus panel list. | Outer context characteristics |
| Bhatia et al 2017[[68](#_ENREF_68)] | Canada | Primary care | Quantitative | Record review | 3 629 859 patients | Resting electrocardiography (ECG) in low-risk patients undergoing an annual health examination | Choosing Wisely | Patient characteristics, Professional characteristics s, Organisational structures related to the LVC, Location |
| Bishop et al 2017[[69](#_ENREF_69)] | USA | Not specified | Qualitative | Focus groups | 24 physicians | LVC in general | Choosing Wisely | Patient expectations, Professional characteristics, Work/care process, Professional, expectations, attitudes and behaviours, Characteristics of the LVC |
| Chan et al 2013[[70](#_ENREF_70)] | USA | Hospital | Quantitative | Record review | 211 254 patients | Inappropriate percutaneous coronary intervention (PCI) for non-acute indications | Appropriate Use Criteria by national cardiovascular societies | Patient characteristics, Location, Economy |
| Charlesworth et al 2016[[71](#_ENREF_71)] | USA | Primary care | Quantitative | Record review | 286 769 Medicaid and  1 376 308 commercial enrollees | Imaging for nonspecific low-back pain  Head imaging for uncomplicated headache  Head imaging for syncope  Imaging for plantar fasciitis  T3 tests for hypothyroidism  Preoperative chest radiography  Abdomen CT  Simultaneous brain and sinus CT  CT for uncomplicated acute rhinosinusitis  Arthroscopic surgery for knee osteoarthritis  Thorax CT  Preoperative echocardiography  Spinal injections for low-back pain  Preoperative stress testing  Preoperative pulmonary function testing  Electroencephalogram for headache | Choosing Wisely, the United Kingdom’s National Institute for Health and Care Excellence | Economy |
| Cho et al 2018[[72](#_ENREF_72)] | Korea | Ambulatory care | Quantitative | Record review | 1 122 080 patients ≥65 years | Central nervous system and psychotropic (CNS-PS) drugs to the Korean elderly population. | Screening Tool of Older Person’s Prescriptions (STOPP criteria) | Setting, Professional characteristics, Health condition |
| Clyne et al 2016[[73](#_ENREF_73)] | Ireland | Primary care | Qualitative | Interviews | 17 General practitioners | Potentially inappropriate prescribing | Beers criteria and Screening Tool of Older Person’s Prescriptions (STOPP criteria) | Work/care process, Patient-provider communication/interaction |
| Colla et al 2018[[74](#_ENREF_74)] | USA | Not specified | Quantitative | Record review | 100 percent Medicare administrative claims data (2009–2011). Commercial and Medicare populations (number not specified) | Early imaging for back pain, vitamin D screening, cervical cancer screening over age 65, prescription opioid use for migraines, cardiac testing in asymptomatic patients, short-interval repeat bone densitometry (DXA), preoperative cardiac testing for low-risk surgery. | Choosing Wisely | Patient characteristics, Economy, Outer context characteristics |
| Dallas et al 2014[[75](#_ENREF_75)] | Australia | Primary care | Qualitative | Survey | 401 General practitioner trainees | Antibiotic prescribing for upper respiratory infections | National guidelines | Patient characteristics, Professional characteristics Professional, expectations, attitudes and behaviours |
| De Rijdt et al 2017[[76](#_ENREF_76)] | Belgium | Hospital | Quantitative | Record review | 597 patients | Inappropriate initiation of long-term acid suppressive therapy in non–critically ill patients | Lanza’s guidelines | Work/care process |
| de Souto Barreto et al 2013[[77](#_ENREF_77)] | France | Aged care | Quantitative | Record review | 6 275 nursing home residents | Long-acting benzodiazepines for older adults | Beers criteria | Patient characteristics, Health condition |
| Dekker et al 2015[[78](#_ENREF_78)] | Netherlands | Primary care | Quantitative | Survey (Registration form) | 2 739 consultations by General practitioners | Inappropriate antibiotics for respiratory tract infections | National Institute for Health and Care Excellence (NICE)  guideline ‘respiratory tract infections-antibiotic prescribing’ | Patient characteristics, Patient expectations, Health condition |
| Di Giorgio et al 2017[[79](#_ENREF_79)] | Italy | Hospital | Quantitative | Record review | 1 027 Patients aged >65 years | Potentially inappropriate drugs | Beers criteria, Screening Tool of Older Person’s Prescriptions/Screening Tool to Alert doctors to Right Treatment criteria, and Improving Prescribing in the Elderly criteria | Health condition |
| Doukky et al 2015[[80](#_ENREF_80)] | USA | Private Community-Based Office Practice | Quantitative | Record review | 1 511 patients | SPECT Myocardial Perfusion Imaging | Appropriate use criteria (AUC) | Economy |
| Ellis et al 2015[[81](#_ENREF_81)] | USA | Hospital | Quantitative | Record review | 12,943 patients | Gonadotropin-releasing Hormone (GnRH) agonist overuse in localized prostate cancer. | The American Urological Association and NCCN clinical practice guidelines | Patient characteristics, Professional characteristics, Staff composition, Economy |
| Extavouret al 2018[[82](#_ENREF_82)] | USA | Office-based practices and community health centers | Mixed methods | Interviews, Survey, Record review | 1 482 practitioners, 32 229 patient records | Potentially inappropriate antidepressant and anxiolytic/sedative prescribing for older, community dwelling adults | Beers Criteria, American Geriatrics Society (AGS) | Patient characteristics, Professional characteristics, Health condition |
| Fleming-Dutra et al 2014[[83](#_ENREF_83)] | USA | Hospital | Quantitative | Survey | 4 178 patient visits | Prescription of broad spectrum antibiotics to children with otitis media. | National guidelines from the American Academy of Paediatrics and American Academy of Family Physicians | Patient characteristics |
| Fonseca et al 2015[[84](#_ENREF_84)] | Portugal | Hospital | Quantitative | Record review | 784 patients | Inappropriate transthoracic echocardiography | Appropriate use criteria (AUC) | Setting, Professional characteristics |
| Grover et al 2016[[85](#_ENREF_85)] | USA | Primary care | Quantitative | Survey | 143 Primary care physicians | Imaging for back pain, Sinusitis treatment, Osteoporosis screening, ECG screening, Pap test and Composite score. | Choosing Wisely | Patient expectations, Work/care process, Professional, expectations, attitudes and behaviours, Evidence, Professional characteristics |
| Hahn et al 2016[[86](#_ENREF_86)] | USA | Hospital | Quantitative | Record review | 6 585 patients with stage 0 to stage II breast cancer | Routine surveillance testing in asymptomatic survivors of early-stage breast cancer | American Society of Clinical Oncology, Choosing Wisely | Setting, Professional characteristics, Health condition |
| Han et al 2013[[87](#_ENREF_87)] | USA | Not specified | Mixed methods | Interviews, Survey, Record review | 1 098 medical oncologists and 980 primary care  physicians | Overuse of surveillance testing for breast cancer survivors | Choosing Wisely, American Society of Clinical Oncology, American College of Physicians | Professional characteristics, Location, Professional, expectations, attitudes and behaviours Evidence,  Patient volume, Setting |
| Hanlon et al 2016[[88](#_ENREF_88)] | USA | Aged care | Quantitative | Record review | 1 303 nursing home patients | Inappropriate prescribing in nursing home patients with dementia. | Beers criteria | Location |
| Chalmers et al 2017[[89](#_ENREF_89)] | UK | Primary care | Quantitative | Record review | 29 815 patients | Inappropriate initial inhaled corticosteroid use in patients with GOLD A/B chronic obstructive pulmonary disease | GOLD guidelines, NICE clinical guidelines for COPD. | Patient characteristics, Setting, Location, Health condition, Time |
| Ramsey et al 2015[[90](#_ENREF_90)] | USA | Not specified | Quantitative | Record review | 39 650 early-stage breast cancer survivors | Inappropriate use of tumor marker  assessment for routine surveillance in non-metastatic breast cancer | American Society of Clinical Oncology, Choosing Wisely | Patient characteristics, Location, Time |
| Selby et al 2018[[91](#_ENREF_91)] | Switzerland | Primary care | Quantitative | Survey | 277 Primary care providers | Imaging for acute low-back pain and long-term  prescribing of proton pump inhibitors | Choosing Wisely | Patient expectations, Work/care process, Professional, expectations, attitudes and behaviours, Interaction between professionals |
| Simos et al 2015[[92](#_ENREF_92)] | Canada | Hospital | Quantitative | Registry data | 26 547 women with early-stage, operable breast cancer | Radiologic imaging for distant metastases in early-stage breast cancer | Choosing Wisely, Cancer Care Ontario guideline, American Society of Clinical Oncology (ASCO). | Patient characteristics. Setting, Health condition |

**Table B.** Information about included studies on determinants for the de-implementation of LVC

| **Reference** | **Country** | **Setting** | **Design** | **Methods** | **Number and type of respondents** | **Low-value care practice** | **Guideline/**  **recommendation** | **Determinants** |
| --- | --- | --- | --- | --- | --- | --- | --- | --- |
| Robert et al 2014[[93](#_ENREF_93)] | International | Policy | Qualitative | Delphi | 30 researchers; policy-makers and  regulators, and; commissioners and providers of healthcare services | Decommission (de-implementation) in general | Not applicable | Evidence, De-implementation process, Policy and political support |
| Silverstein et al 2016[[94](#_ENREF_94)] | Canada | Primary care | Mixed methods | Interviews, Survey | 344 patients in the survey, 54 in the interviews | Annual electrocardiogram testing, imaging for low back pain, the use  of antibiotics for sinusitis, the use of sedative-hypnotics for insomnia, and the use of antipsychotics to treat behavioural symptoms of dementia | The International Choosing Wisely Top 10 list | Patient knowledge |
| Specchia et al 2018[[95](#_ENREF_95)] | Europe | Policy | Quantitative | Survey | Representatives from Ministry of  Health and/or HTA  agencies and/or national institutions | Disinvestment in general and in cancer care in particular | Not applicable | Patient expectations, Professional, expectations, attitudes and behaviours, Evidence, Policy and political support |
| Zikmund-Fisher et al 2017[[96](#_ENREF_96)] | USA | Primary care | Quantitative | Survey | 1 776 primary care physicians and VA primary care providers | 12 Choosing Wisely recommendations | Choosing Wisely recommendations | Patient expectations, Work/care process, Professional, expectations, attitudes and behaviours, Interaction between professionals, Economy |
| Barnes et al 2017[[97](#_ENREF_97)] | USA | Anticoagulation clinic | Mixed methods | Interviews, Survey, | 8 interviews and 40 surveys with nurses and pharmacists | Frequent laboratory testing for patients who are stable on warfarin | Evidence-based management of anticoagulant therapy: Antithrombotic Therapy and Prevention of Thrombosis, 9th ed: American College of Chest Physicians Evidence-Based Clinical Practice Guidelines | Patient expectations, Health condition, Work/care process, De-imp process , Memory |
| Voorn et al 2014[[98](#_ENREF_98)] | Netherlands | Hospital | Mixed methods | Interviews, Survey | 253 orthopaedic surgeons, anaesthesiologists | Erythropoietin and intra- and postoperative blood salvage a in primary elective total hip and knee arthroplasties | Dutch Institute for Healthcare Improvement (CBO). Blood transfusion policy guideline | Policy and political support, Professional, expectations, attitudes and behaviours Pressure from suppliers,  Negative consequences of reducing LVC for the professional,  Characteristics of alternative practice |
| Greene et al 2015[[99](#_ENREF_99)] | USA | Hospital | Quantitative | Survey | 228 emergency medicine residents | Computed tomography scans of the head in  patients with minor head injury who are at low risk | Choosing Wisely | Patient expectations, Professional, expectations, attitudes and behaviours |
| Buist et al 2016[[100](#_ENREF_100)] | USA | Primary care | Quantitative | Survey | 23 physician assistants and 166 physicians | LVC in general | Choosing Wisely | Patient knowledge, Patient expectations, Work/care process, Professional, expectations, attitudes and behaviours |
| Greenwood et al 2017[[101](#_ENREF_101)] | USA | Hospital | Quantitative | Record review | Approximately two million patients | Coronary stents for low-severity stable coronary  arterial disease (SCAD) | Medical guideline by the American Heart Association  and the American College of Cardiology | Setting, Work/care process, Culture, Evidence |

**References**

1. Sharp AL, Klau MH, Keschner D, Macy E, Tang T, Shen E, et al. Low-value care for acute sinusitis encounters: Who's choosing wisely. Am J Manag Care. 2015;21(7):479-85.

2. Scherer LD, Zikmund-Fisher BJ, Fagerlin A, Tarini BA. Influence of "GERD" label on parents' decision to medicate infants. Pediatrics. 2013;131(5):839-45.

3. Weinberg AC, Whalen MJ, Paulucci DJ, Woldu S, Deibert CM, Korets R, et al. Utilization of the Robotic Surgical Platform for Radical Nephrectomy: A National Comparison of Trends for Open, Laparoscopic and Robotic Approaches. Urol Pract. 2016;3(3):187-93.

4. Singh A, Bodukam V, Saigal K, Bahl J, Wang Y, Hanlon A, et al. Identifying Risk Factors Associated with Inappropriate Use of Acid Suppressive Therapy at a Community Hospital. Gastroenterol Res Pract. 2016;2016(Article id:1973086).

5. Schubert I, Küpper-Nybelen J, Ihle P, Thürmann P. Prescribing potentially inappropriate medication (PIM) in Germany's elderly as indicated by the PRISCUS list. An analysis based on regional claims data. Pharmacoepidemiol Drug Saf. 2013;22(7):719-27.

6. Ladapo JA, Blecker S, Douglas PS. Physician decision making and trends in the use of cardiac stress testing in the United States: An analysis of repeated cross-sectional data. Ann Intern Med. 2014;161(7):482-90.

7. Lam PW, Ford BD, Webster C, Zoutman DE. Influences on family physician antibiotic prescribing for uncomplicated urinary tract infection. Can J Infect Control. 2014;29(2):93-8.

8. Rasu RS, Iqbal M, Hanifi SMA, Moula A, Hoque S, Rasheed S, et al. Level, pattern, and determinants of polypharmacy and inappropriate use of medications by village doctors in a rural area of Bangladesh. Clinicoecon Outcomes Res. 2014;6:515-21.

9. Hines JZ, Sewell JL, Sehgal NL, Moriates C, Horton CK, Chen AH. "Choosing wisely" in an academic department of medicine. Am J Med Qual. 2014;30(6):566-70.

10. Sedrak MS, Patel MS, Ziemba JB, Murray D, Kim EJ, Dine CJ, et al. Residents' self-report on why they order perceived unnecessary inpatient laboratory tests. J Hosp Med. 2016;11(12):869-72.

11. Mafi JN, Wee CC, Davis RB, L, on BE. Association of primary care practice location and ownership with the provision of low-value care in the United States. JAMA Intern Med. 2017;177(6):838-45.

12. Nuytten A, Behal H, Duhamel A, Jarreau PH, Mazela J, Milligan D, et al. Evidence-based neonatal unit practices and determinants of Postnatal corticosteroid-use in preterm births below 30 weeks ga in Europe. A population-based cohort study. PLoS ONE. 2017;12(1).

13. Wang DE, Lam DJ, Bellmunt AM, Rosenfeld RM, Ikeda AK, Shin JJ. Intranasal Steroid Use for Otitis Media with Effusion: Ongoing Opportunities for Quality Improvement. Otolaryngol Head Neck Surg. 2017;157(2):289-96.

14. Kerns JW, Winter JD, Winter KM, Boyd T, Etz RS. Primary care physician perspectives about antipsychotics and other medications for symptoms of dementia. J Am Board Fam Med. 2018;31(1):9-21.

15. Redwood R, Knobloch MJ, Pellegrini DC, Ziegler MJ, Pulia M, Safdar N. Reducing unnecessary culturing: A systems approach to evaluating urine culture ordering and collection practices among nurses in two acute care settings. Antimicrob Resist Infect Control. 2018;7(4).

16. Schmidt ML, Spencer MD, Davidson LE. Patient, Provider, and Practice Characteristics Associated with Inappropriate Antimicrobial Prescribing in Ambulatory Practices. Infect Control Hosp Epidemiol. 2018;39(3):307-15.

17. Hong AS, Ross-Degnan D, Zhang F, Wharam JF. Clinician-Level Predictors for Ordering Low-Value Imaging. JAMA Intern Med. 2017;177(11):1577-85.

18. Irfan N, Brooks A, Mithoowani S, Celetti SJ, Main C, Mertz D. A Controlled Quasi-Experimental Study of an Educational Intervention to Reduce the Unnecessary Use of Antimicrobials For Asymptomatic Bacteriuria. PLoS ONE. 2015;10(7):e0132071.

19. Jiron M, Pate V, Hanson LC, Lund JL, Jonsson Funk M, Sturmer T. Trends in Prevalence and Determinants of Potentially Inappropriate Prescribing in the United States: 2007 to 2012. J Am Geriatr Soc. 2016;64(4):788-97.

20. Kachalia A, Berg A, Fagerlin A, Fowler KE, Hofer TP, Flanders SA, et al. Overuse of testing in preoperative evaluation and syncope: a survey of hospitalists. Ann Intern Med. 2015;162(2):100-8.

21. Kachru N, Carnahan RM, Johnson ML, Aparasu RR. Potentially inappropriate anticholinergic medication use in community-dwelling older adults: a national cross-sectional study. Drugs Aging. 2015;32(5):379-89.

22. Kepka D, Breen N, King JB, Meissner HI, R, KB, et al. Demographic factors associated with overuse of Pap testing. Am J Prev Med. 2014;47(5):629-33.

23. Klang E, Beytelman A, Greenberg D, Or J, Guranda L, Konen E, et al. Overuse of Head CT Examinations for the Investigation of Minor Head Trauma: Analysis of Contributing Factors. J Am Coll Radiol. 2017;14(2):171-6.

24. Kondo N, Nakamura F, Yamazaki S, Yamamoto Y, Akizawa T, Akiba T, et al. Prescription of potentially inappropriate medications to elderly hemodialysis patients: prevalence and predictors. Nephrol Dial Transplant. 2014;30(3):498-505.

25. Kruse GR, Khan SM, Zaslavsky AM, Ayanian JZ, Sequist TD. Overuse of colonoscopy for colorectal cancer screening and surveillance. J Gen Intern Med. 2015;30(3):277-83.

26. Kwon AJ, Roshal M, DeSancho MT. Clinical adherence to thrombophilia screening guidelines at a major tertiary care hospital. J Thromb Haemost. 2016;14(5):982-6.

27. Lalude OO, Gutarra MF, Pollono EN, Lee S, Tarwater PM. Inappropriate utilization of SPECT myocardial perfusion imaging on the USA-Mexico border. J Nucl Cardiol. 2014;21(3):544-52.

28. Lim D, Jung J. Racial-Ethnic Differences in Off-Label Antidepressant Use, by Insurance Type. Psychiatr Serv. 2017;68(12):1271-9.

29. Lin MP, Nguyen T, Probst MA, Richardson LD, Schuur JD. Emergency Physician Knowledge, Attitudes, and Behavior Regarding ACEP's Choosing Wisely Recommendations: A Survey Study. Acad Emerg Med. 2017;24(6):668-75.

30. Lipitz-Snyderman A, Sima CS, Atoria CL, Elkin EB, Anderson C, Blinder V, et al. Physician-Driven Variation in Nonrecommended Services Among Older Adults Diagnosed With Cancer. JAMA Intern Med. 2016;176(10):1541-8.

31. Lund BC, Charlton ME, Steinman MA, Kaboli PJ. Regional differences in prescribing quality among elder veterans and the impact of rural residence. Rural Health. 2013;29(2):172-9.

32. Mahal AS, Rhoads KF, Elliott CS, Sokol ER. Inappropriate oophorectomy at time of benign premenopausal hysterectomy. Menopause. 2017;24(8):947-53.

33. Makarov DV, Hu EY, Walter D, Braithwaite RS, Sherman S, Gold HT, et al. Appropriateness of Prostate Cancer Imaging among Veterans in a Delivery System without Incentives for Overutilization. Health Serv Res. 2015;51(3):1021-51.

34. Makarov DV, Soulos PR, Gold HT, Yu JB, Sen S, Ross JS, et al. Regional-Level Correlations in Inappropriate Imaging Rates for Prostate and Breast Cancers: Potential Implications for the Choosing Wisely Campaign. JAMA Oncol. 2015;1(2):185-94.

35. Malo S, Poblador-Plou B, Prados-Torres A, Lallana MJ, Laguna-Berna C, Rabanaque MJ. Poor congruence with guidelines in the use of antibiotics for acute bronchitis: a descriptive study based on electronic health records. Fam Pract. 2016;33(5):471-5.

36. Marchisio P, Tagliabue M, Klersy C, Mira E, Pagella F, Baggi E, et al. Patterns in acute otitis media drug prescriptions: a survey of Italian pediatricians and otolaryngologists. Expert Rev Anti Infect Ther. 2014;12(9):1159-63.

37. Means AR, Weaver MR, Burnett SM, Mbonye MK, Naikoba S, M, et al. Correlates of inappropriate prescribing of antibiotics to patients with malaria in Uganda. PLoS ONE. 2014;9(2):e90179.

38. Moralidis E, Papadimitriou N, Stathaki M, Xourgia X, Spyridonidis T, Fotopoulos A, et al. A multicenter evaluation of the appropriate use of single-photon emission tomography myocardial perfusion imaging in Greece. J Nucl Cardiol. 2013;20(2):275-83.

39. Munoz-Plaza CE, Parry C, Hahn EE, Tang T, Nguyen HQ, Gould MK, et al. Integrating qualitative research methods into care improvement efforts within a learning health system: addressing antibiotic overuse. Health Res Policy Syst. 2016;14(63).

40. Pickles K, Carter SM, Rychetnik L, Entwistle VA. Doctors' perspectives on PSA testing illuminate established differences in prostate cancer screening rates between Australia and the UK: a qualitative study. BMJ Open. 2016;6(12):e011932.

41. Rosenthal MB, Colla CH, Morden NE, Sequist TD, Mainor A, J e, et al. Overuse and insurance plan type in a privately insured population. Am J Manag Care. 2018;24(3):140-6.

42. Ryskina KL, Holmboe ES, Bernabeo E, Werner RM, Shea JA, Long JA. US internists' awareness and use of overtreatment guidelines: a national survey. Am J Manag Care. 2017;23(7):420-7.

43. Sawan M, Jeon Y-H, Fois RA, Chen TF. Exploring the link between organizational climate and the use of psychotropic medicines in nursing homes: a qualitative study. Res Social Adm Pharm. 2017;13(3):513-23.

44. Sawan MJ, Jeon YH, Fois RJ, Chen TF. A qualitative study exploring visible components of organizational culture: what influences the use of psychotropic medicines in nursing homes? Int Psychogeriatr. 2016;28(10):1725-35.

45. Silverman M, Povitz M, Sontrop JM, Li L, Richard L, Cejic S, et al. Antibiotic prescribing for nonbacterial acute upper respiratory infections in elderly persons. Ann Intern Med. 2017;166(11):765-74.

46. Simos D, Hutton B, Graham ID, Arnaout A, Caudrelier JM, Clemons M. Imaging for metastatic disease in patients with newly diagnosed breast cancer: are doctor's perceptions in keeping with the guidelines? J Eval Clin Pract. 2015;21(1):67-73.

47. Tavakoli H, Mark FitzGerald J, Lynd LD, Sadatsafavi M. Predictors of inappropriate and excessive use of reliever medications in asthma: a 16-year population-based study. BMC Pulm Med. 2018;18(33).

48. Undela K, Bansal D, D'Cruz S, Sachdev A, Tiwari P. Prevalence and determinants of use of potentially inappropriate medications in elderly inpatients: a prospective study in a tertiary healthcare setting. Geriatr Gerontol Int. 2014;14(2):251-8.

49. Winchester DE, Hymas J, Meral R, Nguyen D, Dusaj R, Shaw LJ, et al. Clinician-dependent variations in inappropriate use of myocardial perfusion imaging: training, specialty, and location. J Nucl Cardiol. 2013;21(3):598-604.

50. Yaeger JP, Temte JL, Hanrahan LP, Martinez-Donate P. Roles of Clinician, Patient, and Community Characteristics in the Management of Pediatric Upper Respiratory Tract Infections. Ann Fam Med. 2015;13(6):529-36.

51. Chang CB, Lai HY, Yang SY, Wu RS, Liu HC, Hsu HY, et al. Patient- and clinic visit-related factors associated with potentially inappropriate medication use among older home healthcare service recipients. PLoS ONE [Electronic Resource]. 2014;9(4):e94350.

52. Colla CH, Morden NE, Sequist TD, Schpero WL, Rosenthal MB. Choosing wisely: prevalence and correlates of low-value health care services in the United States. J Gen Intern Med. 2015;30(2):221-8.

53. Cuba Fuentes MS, Zegarra Zamalloa CO, Reichert S, Gill D. Attitudes, perceptions and awareness concerning quaternary prevention among family doctors working in the Social Security System, Peru: a cross-sectional descriptive study. Medwave. 2016;16(3):e6433.

54. Botica MV, Botica I, Stamenic V, Andrasevic AT, Kern J, Spehar SS. Antibiotic prescription rate for upper respiratory tract infections and risks for unnecessary prescription in Croatia. Coll Antropol. 2013;37(2):449-54.

55. Faustino CG, Passarelli MC, Jacob-Filho W. Potentially inappropriate medications among elderly Brazilian outpatients. Sao Paulo Med J. 2013;131(1):19-26.

56. Colla CH, Sequist TD, Rosentha MB, Schpero WL, Gottlieb DJ, Morden NE. Use of non-indicated cardiac testing in low-risk patients: Choosing Wisely. BMJ Qual Saf. 2015;24(2):149-53.

57. Cope AL, Francis NA, Wood F, Chestnutt IG. Antibiotic prescribing in UK general dental practice: a cross-sectional study. Community Dent Oral Epidemiol. 2016;44(2):145-53.

58. Grigoryan L, Zoorob R, Shah J, Wang H, Arya M, Trautner BW. Antibiotic Prescribing for Uncomplicated Acute Bronchitis Is Highest in Younger Adults. Antibiotics. 2017;6(4):27.

59. Gieniusz M, Sinvani L, Kozikowski A, Patel V, Nouryan C, Williams MS, et al. Percutaneous Feeding Tubes in Individuals with Advanced Dementia: Are Physicians “Choosing Wisely”? J Am Geriatr Soc. 2018;66(1):64-9.

60. Dempsey PP, Businger AC, Whaley LE, Gagne JJ, Linder JA. Primary care clinicians' perceptions about antibiotic prescribing for acute bronchitis: a qualitative study. BMC Fam Pract. 2014;15:194.

61. Michel-Lepage A, Ventelou B, Nebout A, Verger P, Pulcini C. Cross-sectional survey: risk-averse French GPs use more rapid-antigen diagnostic tests in tonsillitis in children. BMJ Open. 2013;3(e003540).

62. Alber K, Kuehlein T, Schedlbauer A, Schaffer S. Medical overuse and quaternary prevention in primary care - A qualitative study with general practitioners. BMC Fam Pract. 2017;18(1):99.

63. Amos TB, Keith SW, Del Canale S, Orsi P, Maggio M, Baccarini S, et al. Inappropriate prescribing in a large community-dwelling older population: a focus on prevalence and how it relates to patient and physician characteristics. J Clin Pharm Ther. 2015;40(1):7-13.

64. Barlam TF, Morgan JR, Wetzler LM, Christiansen CL, Drainoni ML. Antibiotics for respiratory tract infections: a comparison of prescribing in an outpatient setting. Infect Control Hosp Epidemiol. 2015;36(2):153-9.

65. Barnett ML, Linder JA, Clark CR, Sommers BD. Low-Value Medical Services in the Safety-Net Population. JAMA Intern Med. 2017;177(6):829-37.

66. Bell HT, Steinsbekk A, Granas AG. Factors influencing prescribing of fall-risk-increasing drugs to the elderly: A qualitative study. Scand J Prim Health Care. 2013;33(2):107-14.

67. Beuscart JB, Genin M, Dupont C, Verloop D, Duhamel A, Defebvre MM, et al. Potentially inappropriate medication prescribing is associated with socioeconomic factors: a spatial analysis in the French Nord-Pas-de-Calais Region. Age Ageing. 2017;46(4):607-13.

68. Bhatia RS, Bouck Z, Ivers NM, Mecredy G, Singh J, Pendrith C, et al. Electrocardiograms in Low-Risk Patients Undergoing an Annual Health Examination. JAMA Intern Med. 2017;177(9):1326-33.

69. Bishop TF, Cea M, Miranda Y, Kim R, Lash-Dardia M, Lee JI, et al. Academic physicians' views on low-value services and the choosing wisely campaign: A qualitative study. Healthcare. 2017;5(1-2):17-22.

70. Chan PS, Rao SV, Bhatt DL, Rumsfeld JS, Gurm HS, Nallamothu BK, et al. Patient and hospital characteristics associated with inappropriate percutaneous coronary interventions. J Am Coll Cardiol. 2013;62(24):2274-81.

71. Charlesworth CJ, Meath TH, Schwartz AL, McConnell KJ. Comparison of low-value care in Medicaid vs commercially insured populations. JAMA Intern Med. 2016;176(7):998-1004.

72. Cho H, Choi J, Kim YS, Son SJ, Lee KS, Hwang HJ, et al. Prevalence and predictors of potentially inappropriate prescribing of central nervous system and psychotropic drugs among elderly patients: A national population study in Korea. Arch Gerontol Geriatr. 2018;74:1-8.

73. Clyne B, Cooper JA, Hughes CM, Fahey T, Smith SM, team O-Ss. 'Potentially inappropriate or specifically appropriate?' Qualitative evaluation of general practitioners views on prescribing, polypharmacy and potentially inappropriate prescribing in older people. BMC Fam Pract. 2016;17(1):109.

74. Colla CH, Morden NE, Sequist TD, Mainor AJ, Li Z, Rosenthal MB. Payer Type and Low-Value Care: Comparing Choosing Wisely Services across Commercial and Medicare Populations. Health Serv Res. 2018;53(2):730-46.

75. Dallas A, Magin P, Morgan S, Tapley A, Henderson K, Ball J, et al. Antibiotic prescribing for respiratory infections: a cross-sectional analysis of the ReCEnT study exploring the habits of early-career doctors in primary care. Fam Pract. 2014;32(1):49-55.

76. De Rijdt T, Spriet I, Willems L, Blanckaert M, Hiele M, Wilmer A, et al. Appropriateness of Acid Suppression Therapy. Ann Pharmacother. 2017;51(2):125-34.

77. de Souto Barreto P, Lapeyre-Mestre M, Mathieu C, Piau C, Bouget C, Cayla F, et al. Indicators of benzodiazepine use in nursing home residents in France: a cross-sectional study. J Am Med Dir Assoc. 2013;14(1):29-33.

78. Dekker AR, Verheij TJ, van der Velden AW. Inappropriate antibiotic prescription for respiratory tract indications: most prominent in adult patients. Fam Pract. 2015;32(4):401-7.

79. Di Giorgio C, Provenzani A, Polidori P. Potentially inappropriate drug prescribing in elderly hospitalized patients: an analysis and comparison of explicit criteria. Int J Clin Pharm. 2017;38(2):462-8.

80. Doukky R, Hayes K, Frogge N, Nazir NT, Collado FM, Williams KA, Sr. Impact of insurance carrier, prior authorization, and socioeconomic status on appropriate use of SPECT myocardial perfusion imaging in private community-based office practice. Clin Cardiol. 2015;38(5):267-73.

81. Ellis SD, Nielsen ME, Carpenter WR, Jackson GL, Wheeler SB, Liu H, et al. Gonadotropin-releasing hormone agonist overuse: urologists' response to reimbursement and characteristics associated with persistent overuse. Prostate Cancer Prostatic Dis. 2015;18(2):173-81.

82. Extavour RM, Perri M, 3rd. Patient, Physician, and Health-System Factors Influencing the Quality of Antidepressant and Sedative Prescribing for Older, Community-Dwelling Adults. Health Serv Res. 2018;53(1):405-29.

83. Fleming-Dutra KE, Shapiro DJ, Hicks LA, Gerber JS, Hersh AL. Race, otitis media, and antibiotic selection. Pediatrics. 2014;134(6):1059-66.

84. Fonseca P, Sampaio F, Ribeiro J, Goncalves H, Gama V. Appropriate use criteria for transthoracic echocardiography at a tertiary care center. Rev Port Cardiol. 2015;34(12):713-8.

85. Grover M, Abraham N, Chang YH, Tilburt J. Physician Cost Consciousness and Use of Low-Value Clinical Services. J Am Board Fam Med. 2016;29(6):785-92.

86. Hahn EE, Tang T, Lee JS, Munoz‐Plaza CE, Shen E, Rowley B, et al. Use of posttreatment imaging and biomarkers in survivors of early‐stage breast cancer: Inappropriate surveillance or necessary care? Cancer. 2016;122(6):908-16.

87. Han PK, Klabunde CN, Noone AM, Earle CC, Ayanian JZ, Ganz PA, et al. Physicians' beliefs about breast cancer surveillance testing are consistent with test overuse. Med Care. 2013;51(4):315-23.

88. Hanlon JT, Aspinall SL, H, ler SM, Gellad WF, Stone RA, et al. Potentially suboptimal prescribing for older veteran nursing home patients with dementia. Ann Pharmacother. 2015;49(1):20-8.

89. Chalmers JD, Tebboth A, Gayle A, Ternouth A, Ramscar N. Determinants of initial inhaled corticosteroid use in patients with GOLD A/B COPD: a retrospective study of UK general practice. NPJ Prim Care Respir Med. 2017;27(1):1-8.

90. Ramsey SD, Henry NL, Gralow JR, Mirick DK, Barlow W, Etzioni R, et al. Tumor marker usage and medical care costs among older early-stage breast cancer survivors. J Clin Oncol. 2015;33(2):149-55.

91. Selby K, Cornuz J, Cohidon C, Gaspoz J-M, Senn N. How do Swiss general practitioners agree with and report adhering to a top-five list of unnecessary tests and treatments? Results of a cross-sectional survey. Eur J Gen Pract. 2018;24(1):32-8.

92. Simos D, Catley C, van Walraven C, Arnaout A, Booth CM, McInnes M, et al. Imaging for distant metastases in women with early-stage breast cancer: a population-based cohort study. CMAJ. 2015;187(12):E387-E97.

93. Robert G, Harlock J, Williams I. Disentangling rhetoric and reality: an international Delphi study of factors and processes that facilitate the successful implementation of decisions to decommission healthcare services. Implement Sci. 2014;9(123).

94. Silverstein W, Lass E, Born K, Morinville A, Levinson W, Tannenbaum C. A survey of primary care patients' readiness to engage in the de-adoption practices recommended by Choosing Wisely Canada. BMC Res Notes. 2016;9(301).

95. Specchia ML, La Torre G, Calabro GE, Villari P, Grilli R, Federici A, et al. Disinvestment in cancer care: a survey investigating European countries' opinions and views. Eur J Public Health. 2018;28(6):987-2.

96. Zikmund-Fisher BJ, Kullgren JT, Fagerlin A, Klamerus ML, Bernstein SJ, Kerr EA. Perceived Barriers to Implementing Individual Choosing Wisely® Recommendations in Two National Surveys of Primary Care Providers. J Gen Intern Med. 2017;32(2):210-7.

97. Barnes GD, Misirliyan S, Kaatz S, Jackson EA, Haymart B, Kline-Rogers E, et al. Barriers and facilitators to reducing frequent laboratory testing for patients who are stable on warfarin: a mixed methods study of de-implementation in five anticoagulation clinics. Implement Sci. 2017;12(87).

98. Voorn VM, Marang‐van de Mheen PJ, Wentink MM, Kaptein AA, Koopman‐van Gemert AW, So‐Osman C, et al. Perceived barriers among physicians for stopping non–cost‐effective blood‐saving measures in total hip and total knee arthroplasties. Transfusion. 2014;54(10pt2):2598-607.

99. Greene SE, Massone R. A survey of emergency medicine residents' perspectives of the choosing wisely campaign. Am J Emerg Med. 2015;33(6):853-5.

100. Buist DS, Chang E, Handley M, Pardee R, Gundersen G, Cheadle A, et al. Primary Care Clinicians' Perspectives on Reducing Low-Value Care in an Integrated Delivery System. Perm J. 2016;20(1):41-6.

101. Greenwood BN, Agarwal R, Agarwal R, Gopal A. The When and Why of Abandonment: The Role of Organizational Differences in Medical Technology Life Cycles. Manage Sci. 2016;63(9):2948-66.
